# Powerful or non-powerful? Revisiting hallucal grasping as a key evolutionary innovation in small arboreal mammals

**DOI:** 10.1016/j.isci.2026.115913

**Published:** 2026-04-29

**Authors:** Irene Montañez-Rivera, Séverine L.D. Toussaint, Alexander Stoessel, Armita R. Manafzadeh, Ute Radespiel, Vera Bruhn, Marie Newbon, John A. Nyakatura

**Affiliations:** 1 Comparative Zoology, Institut für Biologie, Humboldt Universität zu Berlin, Berlin, Germany; 2 Functional Morphology, Institute of Biology, University of Antwerp, Antwerp, Belgium; 3 UMR 7207-CR2P, CNRS/MNHN/Sorbonne Université, Paris, France; 4 Institute of Zoology and Evolutionary Research, Friedrich Schiller University Jena, Jena, Germany; 5 Department of Archaeogenetics, Max Planck Institute for Evolutionary Anthropology, Leipzig, Germany; 6 Yale Institute for Biospheric Studies, Yale University, New Haven, CT, USA; 7 Department of Earth and Planetary Sciences, Yale University, New Haven, CT, USA; 8 Yale Peabody Museum of Natural History, New Haven, CT, USA; 9 Stiftung Tierärztliche Hochschule Hannover, Hannover, Germany

**Keywords:** Wildlife anatomy, Zoology, Evolutionary biology

## Abstract

Powerful hallucal grasping is considered a key primate innovation, yet many arboreal mammals show convergent pedal grasping. We hypothesized that hallucal grasping represents a functional continuum beyond the dichotomy of powerful versus non-powerful grasping. Using diceCT to analyze intrinsic hallucal muscle architecture and automated quantification of the entocuneiform-first metatarsal joint (EFMJ) range of motion, we modeled muscle moment arms in 13 small arboreal mammals (primates, scandentians, rodents, and marsupials). All taxa feature a saddle-shaped EFMJ but differ in kinematical emphasis: primates and marsupials favor flexion-extension through facet torsion, enlarged peroneal processes or tilted articulation surfaces, whereas scandentians and rodents prioritize abduction-adduction. Strepsirrhine primates and marsupials possess enlarged flexors and strong adductors enabling sustained torque, while scandentians and rodents are characterized by low anatomical cross-sectional area muscles. Moment-arm analyses reveal torque optimization at different poses, highlighting a spectrum of morphofunctional grasping strategies rather than a single evolutionary pathway.

## Highlights

• Morpho-functional analyses reveal differences in hallucal grasping adaptations
• Among small arboreal mammals, primates and marsupials show increased hallux mobility
• Tupaia and rodents rely on lower cross-sectional intrinsic muscles
• All species optimize torque at different entocuneiform-metatarsal joint poses

## Graphical abstract

**Figure:**
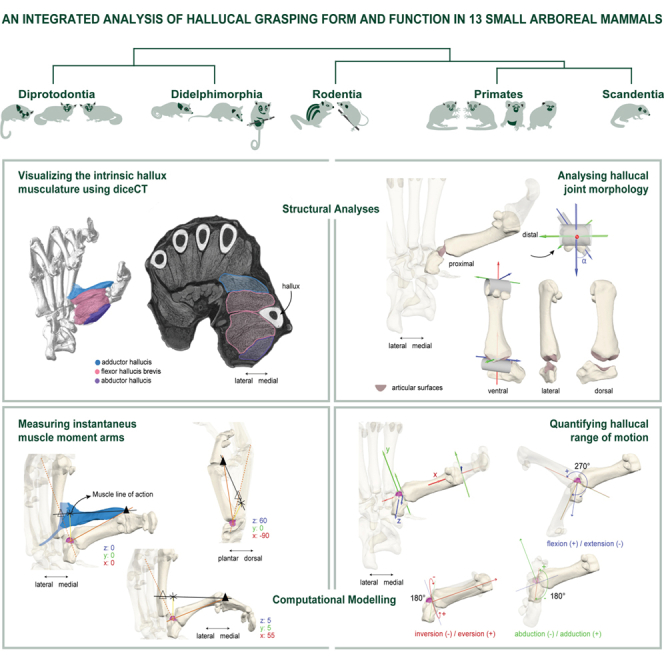

## Introduction

**Figure 1:**
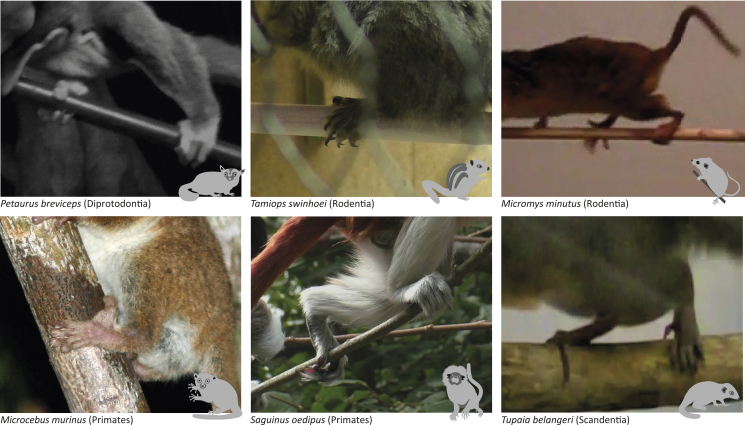
Photographs showing examples of hallucal grasping in various primate and non-primate mammals during quadrupedal locomotion, climbing, or resting on several support sizes

The ability for hallucal pedal grasping is considered a key innovation in early primate evolution and is characterized by a highly diverging first digit (hallux) with a flat nail instead of a sharp claw, and elongated phalanges on the lateral digits.^1^^,^^2^^,^^3^^,^^4^^,^^5^ These features are considered specializations for arboreal locomotion and associated lifestyle and are shared by all primate species (except bipedal humans) and their last common ancestor.^6^^,^^7^^,^^8^^,^^9^^,^^10^^,^^11^^,^^12^ Hallucal grasping is the most common foot grasping posture in extant primates and consists of placing the support between digit 1 (hallux) and the lateral digits.^9^^,^^13^ In the literature, there is a tendency to describe the primate hallucal grasp as “powerful,”^14^^,^^15^^,^^16^^,^^17^^,^^18^ and the grasp of most non-primate species as “non-powerful” (or some analogue indicating lack of power).^18^^,^^19^^,^^20^^,^^21^ However, arboreal specialists exist in multiple lineages of eutherian and metatherian mammals and studies on mammalian foot postures during locomotion have highlighted that long-standing views regarding the functional significance of hallucal postures are worth revisiting^13^^,^^22^^,^^23^^,^^24^ (Figure 1). Non-primates, including scandentians but also marsupials and rodents, have been observed to nimbly navigate their environment, engaging in secure hallucal grasp during locomotion in different support types despite their possession of claws and a morphologically less divergent hallux,^13^^,^^19^^,^^21^^,^^22^^,^^24^^,^^25^^,^^26^^,^^27^ and even support their body weight through this grasp while freeing the forelimbs.^13^^,^^28^

The number of behavioral and mechanical studies that support the ability of powerful grasping in non-primate arboreal mammals has increased, with evidence in both marsupial (such as *Caluromys philander*^13^^,^^23^^,^^26^^,^^27^^,^^29^) and rodent species.^28^^,^^30^ Powerful hallucal grasping in non-primates, and specifically in some arboreal marsupials, has been proposed to be a convergent hallucal postural adaptation regarding primates.^23^^,^^31^^,^^32^ Most studies that have investigated the hallucal grasping “power” of arboreal models rely on biomechanical quantification of the mean support reaction forces of the limbs during locomotion or maximal manual and pedal pull strength capacity in primates and other rare models,^15^^,^^33^^,^^34^ but do not account for the actual force-generating capacity of the hallucal intrinsic musculature. “Power” is a term often used as a binary character, although a range of relative force-generating capacity can be expected when comparing species with different foot anatomy and morphology.^23^^,^^35^ It remains unclear to what extent primates and other arboreal specialists might share an adaptation for powerful hallucal grasping capability both at the muscular and osteological scales.

Non-primate arboreal mammals are morphologically adapted to the functional and mechanical demands that are imposed by the tree environment, proving that successful arboreal existence is also possible without primate-like characteristics. It is very likely that pedal grasping and associated hallucal use are early adaptations in mammals that have evolved convergently multiple times.^13^^,^^18^^,^^21^^,^^22^^,^^23^^,^^26^^,^^31^^,^^32^^,^^36^^,^^37^^,^^38^^,^^39^ For arboreal mammals in general, effective hallucal grasping is thought to be achieved by hindfeet with powerful extrinsic flexors with long tendons, independent movement of the digits, extended mobility of the ankle and of intertarsal joints, as well as soft, sensitive and expanded foot pads with excretory glands at high densities, all contributing to a prehensility characterized by increased friction, interlocking, and bonding with the support.^13^^,^^40^^,^^41^^,^^42^^,^^43^^,^^44^ But to date, the function of the intrinsic foot musculature in relation to grasping abilities in non-primate mammals has received little attention.

Fortunately, more detailed descriptions are available for primate hallucal musculoskeletal adaptations. Primate hallucal grasping is accomplished by flexion and adduction of the first metatarsus at the saddle-shaped entocuneiform-first metatarsal joint (EFMJ).^45^ The first metatarsal exhibits a distal epiphysis laterally rotated and a shallow and broad distal phalanx with a flattened nail.^14^^,^^46^ Furthermore, primates achieve grasping at a muscular level by the combined action of their extrinsic and intrinsic foot muscles, which may vary slightly in number and position among species but fall within a common bauplan.^9^^,^^45^^,^^47^ The extrinsic flexor hallucis longus and flexor digitorum longus have muscle bellies located in the lower leg that flex the hallux and the lateral digits, respectively, via long tendons. The intrinsic foot muscles originate within the foot and insert on all digits, with the hallux supplied specifically by the flexor hallucis brevis and adductor hallucis (both involved in grasping), as well as the abductor hallucis and extensor hallucis brevis.^45^

In this study, we investigate the morpho-functional mechanism of hallucal grasping by the combined examination of the intrinsic muscular anatomy and architecture, the EFMJ range of motion (ROM), and instantaneous muscle moment arms (IMMAs) of the intrinsic hallux musculature in a phylogenetically diverse sample of small arboreal mammals. Considering the importance of body size in relation to support size in the mechanics of arboreal locomotion, and the most likely small body size of both the euarchontan and the euprimate ancestors,^18^^,^^35^^,^^48^^,^^49^ we analyzed 13 species with small body sizes: four primates (two strepsirrhines and two platyrrhines), two rodents, one scandentian, and six marsupials (three diprotodontians and three didelphimorphs).

Anatomical features of the musculature, such as the presence or absence of muscles, attachment sites and spatial arrangement determine its function and reflect the ecological adaptations of species.^50^^,^^51^ Muscular architecture, the macroscopic arrangement of muscle fibers relative to the axis of its force generation, determines the muscle’s performance, i.e., the force generation capacity, maximum isometric force, and excursion capability, and is largely consistent between individuals of the same species.^52^^,^^53^ The understanding of muscular function and performance through the analysis of muscular architecture has yielded important knowledge of the constraints that determine the architectural properties and of the species’ anatomical adaptations to different locomotor behaviors.^50^^,^^54^ Although the pedal muscle anatomy of several arboreal taxa has been well documented, its evaluation within a functional and comparative framework that integrates muscle architecture remains necessary (e.g., Oishi et al.,^55^ for two Hominidae, and Gebo,^9^ that quantified the mass of, among others, the adductor hallucis in relation to the total mass of the intrinsic foot muscles of *Microcebus*), in order to better understand primate and non-primate muscular adaptations to grasping. Under the “muscle architecture hypothesis” (H1), we test whether intrinsic hallucal muscles of primates and arboreal marsupials, which have been proposed to exhibit convergent hallucal grasping,^26^^,^^29^^,^^31^ exhibit anatomical capacities that confer them greater force-generation capacities compared to other arboreal mammals.

The 3D osteological ROM of the hallux reflecting the digit’s overall mobility is determined by the morphology of the EFMJ and, to our knowledge, has not been investigated quantitatively before in non-human mammals. The mobility of the hallux is intrinsically correlated with pedal grasping capabilities, and a greater abduction of the hallux has been suggested as a defining component of the “powerful grasping” in primates and arboreal marsupials.^18^^,^^23^^,^^46^ According to our “osteological mobility hypothesis” (H2), we test whether primates and marsupials are capable of a greater three-dimensional ROM at the EFMJ compared to other arboreal mammals.

The IMMA is the perpendicular and thus shortest distance between the muscle’s line of action (MLOA) and the joint’s center of rotation (COR) at each joint pose. Moment arms transform the linear forces developed by muscles into rotational moments that result in joint movements: the product of the muscle force acting along the MLOA and the length of the muscle moment arm defines the torque generated by the muscle.^56^^,^^57^^,^^58^^,^^59^ Large IMMAs facilitate torque, while small IMMAs benefit fast joint excursion, reflecting ecomorphological adaptations in mammals.^60^^,^^61^ Thus, by modeling IMMAs, which are influenced by muscle anatomy, topography, and joint morphology, we can infer mechanical advantage at hallucal positions according to their ROM indicating grasping specialization. Although IMMAs have been examined in relation to leg muscles in some primates and rodents, they remain understudied in the foot in most arboreal species.^59^^,^^62^^,^^63^^,^^64^ Under our “moment arm hypothesis” (H3), we test whether primates possess greater IMMAs for the hallucal adductor and for the hallucal flexor compared to other arboreal species except for arboreal marsupials, that might convergently share comparable IMMAs indicating greater torque production and mechanical advantage during hallucal grasping.

**Figure 2:**
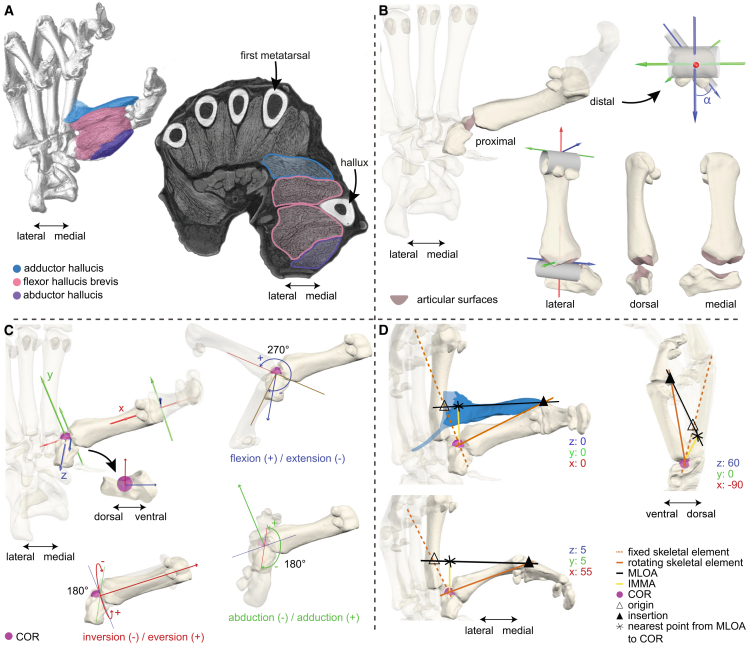
Methodology employed to examine the muscular and osteological mechanisms of hallucal grasping exemplified in the marsupial *Gracilinanus* sp. MfN_GR01 (A) Model in plantar view and cross-section in distal view showing the hallucal muscular anatomy extracted from diceCT scans. (B) Qualitative assessment of the tarso-metatarsal joint morphology and degree of torsion of the metatarsal about its long axis (α). (C) Osteological ROM determined by simultaneously calculating motion in *z*, *y*, and *x* planes to determine viable joint poses within indicated ranges. (D) IMMAs illustrated for the adductor hallucis in the ground position and two viable positions indicated by *zyx* coordinates. COR, center of rotation; MLOA, muscle line of action.

To test our predictions, we visualized and quantified fine-scale anatomical structures (bones and soft tissues) using diceCT^65^ and combined the extracted data to model the musculoskeletal function of the intrinsic hallucal musculature during grasping (Figure 2).

## Results

A detailed description of the muscular anatomy, and further information on muscular architectural properties, joint morphology, ROM, and IMMAs can be found in the supplemental materials (Tables S1, S2, and S3; Figures S1–S6; Data S1). We here focus on the most crucial results in light of the research questions.

### Muscular properties

**Figure 3:**
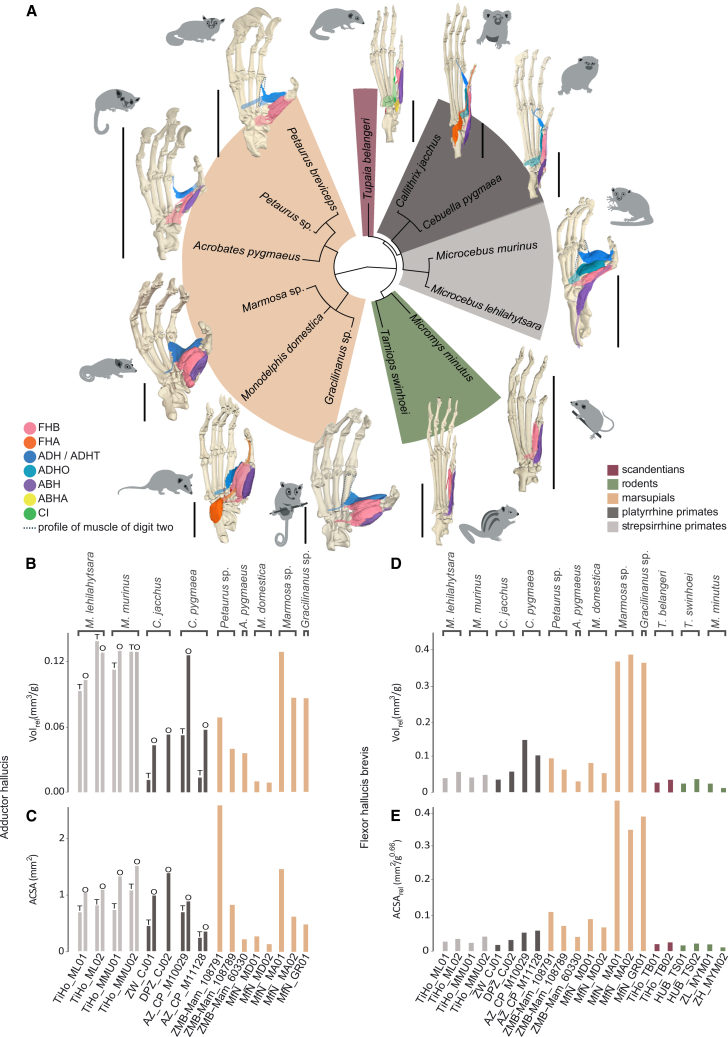
Muscular properties (A) 3D musculoskeletal models showing the muscular topology of the intrinsic hallux-related plantar musculature of the right foot of one individual of each genus, and their associated phylogenetic relationships (obtained from http://timetree.org^66^). The muscles shown are flexor hallucis brevis (FHB), flexor hallucis accessorius (FHA), adductor hallucis (ADH), adductor hallucis transverse head (ADHT), adductor hallucis oblique head (ADHO), abductor hallucis (ABH), abductor hallucis accessorius (ABHA), and contrahens I (CI). Scale bars, 10 mm. (B) Relative volume and (C) ACSA of the adductor hallucis with separate measurements of transverse (T) and oblique (O) heads (if present). (D) Relative volume and (E) relative ACSA of the flexor hallucis brevis. Both muscles are functionally crucial in the hallucal grasp mechanism. See also Tables S1 and S2.

Our diceCT-based investigation of the hallucal morphology of 22 individuals from 13 species of small mammals (Table 1; Figure 2A) revealed that species-specific intrinsic muscular complexes associated with the hallux vary in the number of muscles, topologies, and architectural parameters (Figure 3; Tables S1 and S2). We found that the relative volume and anatomical cross-sectional area (ACSA) of the oblique and transverse heads of adductor hallucis are generally larger in the strepsirrhine primates studied (*Microcebus*) than in the platyrrhine primates, and are variable for the adductor hallucis depending on the species within marsupials. In primates, the oblique head of the adductor hallucis arises from the tendinous sheet plantar to the entocuneiform or the proximal aspect of metatarsal I and attaches to the metatarsal’s lateral sesamoid bone. The transverse head in primates is associated with metatarsals and muscles of digits II and/or III in its origin and extends “transversely” toward digit I. The marsupials display one adductor hallucis muscle with topology comparable to the transverse head of the primate adductor hallucis. In *C. jacchus* DPZ_CJ02, only the oblique head is present, while *T. belangeri* and rodents lack an adductor hallucis.

**Table 1:** List of specimens studied

	Family	Species	Specimen number	Body mass (g)	Sex	Age	Scanner	Voxel size (μm)
Strepsirrhini	Cheirogaleidae	*Microcebus lehilahytsara*	TiHo_ML01	48.00	m	8 years 1 m	A	3.0
Strepsirrhini	Cheirogaleidae	*Microcebus lehilahytsara*	TiHo_ML02	43.40	m	4 years 1 m	A	3.5
Strepsirrhini	Cheirogaleidae	*Microcebus murinus*	TiHo_MMU01	54.90	m	6 years 6 m	A	3.3
Strepsirrhini	Cheirogaleidae	*Microcebus murinus*	TiHo_MMU02	74.00	f	8 years 9 m	B	6.0
Platyrrhini	Callitrichidae	*Callithrix jacchus*	ZW_CJ01	255.00	f	10 years	C	11.0
Platyrrhini	Callitrichidae	*Callithrix jacchus*	DPZ_CJ02	284.00	f	9 years	C	11.0
Platyrrhini	Callitrichidae	*Cebuella pygmaea*	AZ_CP_M10029	60.00	f	2 years	A	3.2
Platyrrhini	Callitrichidae	*Cebuella pygmaea*	AZ_CP_M11128	49.40	m	4 years 11 m	A	3.5
Scandentia	Tupaiidae	*Tupaia belangeri*	TiHo_TB01	196.80	f	6 years 5 m	D	9.0
Scandentia	Tupaiidae	*Tupaia belangeri*	TiHo_TB02	175.00	m	5 years 7 m	C	6.0
Rodentia	Sciuridae	*Tamiops swinhoei*	HUB_TS01	119.80	m	6 years	A	5.1
Rodentia	Sciuridae	*Tamiops swinhoei*	HUB_TS02	56.74	m	unknown	A	7.0
Rodentia	Muridae	*Micromys minutus*	ZL_MYM01	7.91	m	5 m	A	2.6
Rodentia	Muridae	*Micromys minutus*	ZH_MYM02	6.14	m	1 year 6 m	E	4.5
Diprotodontia	Petauridae	*Petaurus* sp.	ZMB-Mam_108791	120.31	unknown	unknown	A	3.6
Diprotodontia	Petauridae	*Petaurus breviceps*	ZMB-Mam_108789	97.50	f	unknown	C	7.0
Diprotodontia	Acrobatidae	*Acrobates pygmaeus*	ZMB-Mam_60330	11.44	unknown	unknown	C	4.0
Didelphimorphia	Didelphidae	*Monodelphis domestica*	MfN_MD01	128.10	m	2 years 5 m	A	3.5
Didelphimorphia	Didelphidae	*Monodelphis domestica*	MfN_MD02	68.29	f	1 year 4 m	A	3.5
Didelphimorphia	Didelphidae	*Marmosa* sp.	MfN_MA01	61.40	unknown	unknown	C	7.0
Didelphimorphia	Didelphidae	*Marmosa* sp.	MfN_MA02	42.89	unknown	unknown	C	7.0
Didelphimorphia	Didelphidae	*Gracilinanus* sp.	MfN_GR01	22.50	unknown	unknown	C	5.0

Specimens were collected from the following institutions: AZ, Antwerp Zoo (Royal Zoological Society of Antwerp), Belgium; DPZ, Deutsches Primatenzentrum, Göttingen, Germany; HUB, Humboldt-Universität zu Berlin, Germany; TiHo, Stiftung Tierärztliche Hochschule Hannover, Germany; ZH, Zoo Halle, Germany; ZL, Zoo Landau, Germany; ZMB, MfN, Museum für Naturkunde Berlin, Germany; ZW, Zoo in der Wingst, Germany. Scanners used: A—SkyScan 2211 (Bruker Corporation, Billerica, MA, USA) at the Max Planck Institute for Evolutionary Anthropology, Leipzig, Germany; B—SkyScan 2214 (Bruker Corporation, Billerica, MA, USA) at Prüflabor Bruker in Karlsdorf-Neuthard, Germany; C—YXLON FF20 (YXLON International GmbH) in Hamburg, Germany; D—Zeiss Xradia Context (Carl Zeiss Microscopy GmbH) in Jena; E—YXLON FF20 CT (YXLON International GmbH) at Humboldt-Universität zu Berlin, Germany.

For all marsupials, the relative volume of the adductor hallucis is lower than for the transverse head of this muscle in *Microcebus*, except in *Marmosa* sp. MfN_MA01 (Figure 3B). However, compared to platyrrhine primates, values are higher in *Petaurus* sp. ZMB-Mam_108791, *Gracilinanus* sp. MfN_GR01, and both specimens of *Marmosa* sp., while the species *Monodelphis domestica* shows the lowest values of the sample. The adductor hallucis in some specimens shows larger ACSA than the transverse head in all primates, like *Marmosa* sp. MfN_MA01 and *Petaurus* sp. ZMB-Mam_108791 with the largest ACSA of all specimens (Figure 3C). *M. domestica* MfN_MD02 and *A. pygmaeus* show the lowest ACSA values, below both *Microcebus* and platyrrhines. The oblique head in primates is generally predominant over the transverse head, with a larger relative volume and ACSA. In *C. jacchus* DPZ_CJ02, the oblique head has a relatively high ACSA comparable to that of *Microcebus*. In all primate individuals, the oblique head of the adductor hallucis has a larger ACSA than the other muscles, except for *C. pygmaea* AZ_CP_M11128, where the flexor hallucis brevis has a larger value.

Moreover, we found that the flexor hallucis brevis muscles in the marsupials *Marmosa* and *Gracilinanus* have the largest relative ACSA of the dataset, over 3 times larger than in *Petaurus* sp. ZMB-Mam_108791 with the next largest ACSA. A similar trend is observed for the relative volume of flexor hallucis brevis, except that both callitrichine specimens of *C. pygmaea* have higher volumes than the diprotodontian and *M. domestica* marsupials, but also higher than the rest of the primate species investigated (Figure 3D). Most marsupials have larger relative ACSA for the flexor hallucis brevis than *Microcebus* and the platyrrhines that show similar values, and *T. belangeri* and rodents that show the lowest values (Figure 3E). The flexor hallucis brevis is present in all specimens, varying in origin among taxa between tarsals (navicular, cuneiforms, and cuboid), metatarsal I, and the tendinous sheet plantar to the cuneiforms. The insertions vary from the medial sesamoid of the first metatarsal in primates, the lateral sesamoid in *T. belangeri* and the rodents, and both sesamoids in the marsupials. In *P. breviceps* ZMB-Mam_108789 and the didelphimorphs, this muscle consists of two bellies that fuse in their course. In all marsupials, the flexor hallucis brevis has a higher ACSA than all other muscles, except in *A. pygmaeus* ZMB-Mam_60330 and *Petaurus* sp. ZMB-Mam_108791, in which the value for the adductor hallucis is slightly higher.

Additionally, we observed that the flexor hallucis accessorius, abductor hallucis, abductor hallucis accessorius, contrahens I, and extensor hallucis brevis are present in different taxa. The flexor hallucis accessorius is present in *C. jacchus* and *M. domestica* and arises from the calcaneum in both. In the first, where the long flexors do not supply the hallux, it attaches at the distal hallucal phalanx, while in the second, it merges through a tendon with that of the long flexors. In *C. jacchus*, the flexor hallucis accessorius muscles have relative volumes (0.035 mm^3^/g in ZW_CJ0 and 0.057 mm^3^/g in DPZ_CJ02) larger than in *M. domestica* (0.015 mm^3^/g in MfN_MD01 and 0.008 mm^3^/g in MfN_MD02), and a relative ACSA (0.017 mm^2^/g^0.66^ in ZW_CJ01 and 0.030 mm^2^/g^0.66^ in DPZ_CJ02) lower than in *M. domestica* (0.022 mm_2_/g^0.66^ in MfN_MD01 and 0.067 mm^2^/g^0.66^ in MfN_MD02).

The foot of *T. belangeri* is the only one with a plantar bony plaque, an abductor accessorius and contrahens I muscle. The latter is part of a complex of three muscles inserting into digits I, II, and V, has a relative volume of 0.006 mm^3^/g in TiHo_TB01 and of 0.009 mm^3^/g in TiHo_TB02, an ACSA of 0.16 mm^2^, and a relative ACSA of 0.005 mm^2^/g^0.66^ in both specimens.

The abductor hallucis is present in all specimens except *Petaurus*, and the extensor hallucis brevis, which is the only dorsal intrinsic muscle, is present in *T. belangeri*, *T. swinhoei*, *M. domestica*, *Marmosa* and *C. jacchus ZW_CJ01.* However, these muscles are not discussed further, as their role in powerful grasping is not considered relevant.

### Osteological properties and ROM

**Figure 4:**
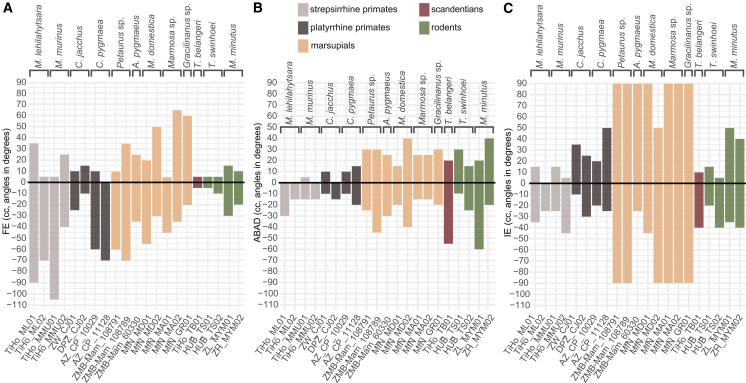
Visualization of the minimal and maximal cosine-corrected (cc) ROM in the ectocuneiform-metatarsal joint for each individual studied Degrees of (A) hallucal flexion-extension (FE), (B) abduction-adduction (ABAD), and (C) inversion-eversion (IE) during combined motion about the three axes. Positive angles indicate hallucal flexion, adduction and eversion, and negative angles indicate hallucal extension, abduction and inversion, respectively. Angles in degrees.

**Figure 5:**
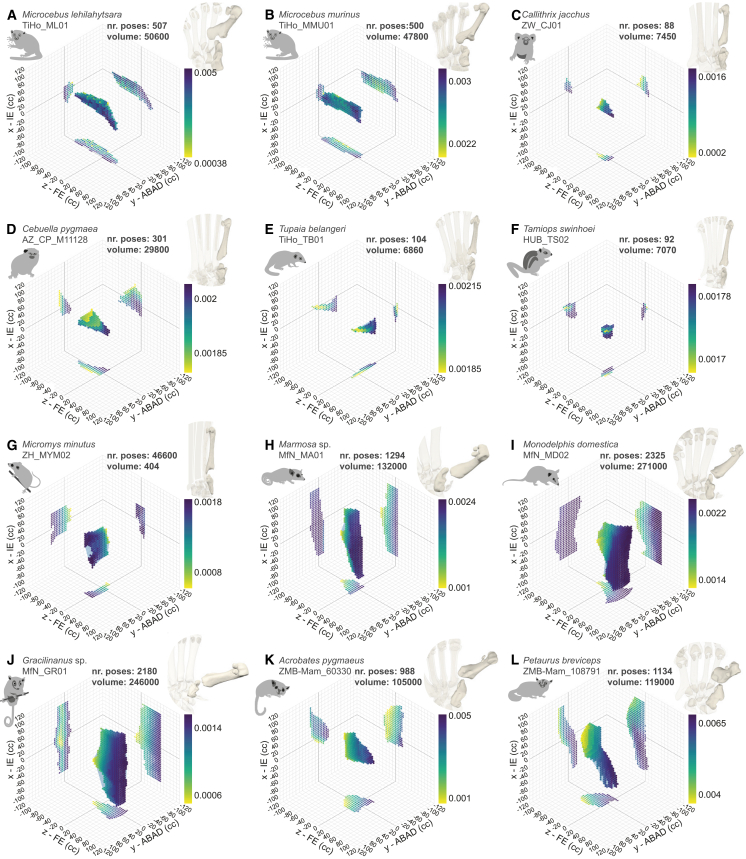
Visualization of the EFMJ of example specimens, ROM at this joint, and IMMAs for the flexor hallucis brevis (A-L) Alpha shapes represent cosine-corrected (cc) ROM with interaction of the three rotational degrees of freedom and points representing viable poses. The *x*, *y*, and *z* axes represent inversion-eversion (IE), abduction-adduction (ABAD), and flexion-extension (FE), respectively. The volume of the alpha shapes is in cubic degrees, and larger volumes indicate increased hallucal mobility. The IMMA (unitless) calculated for the flexor hallucis brevis at each pose is color-coded using individual scales. Poses with higher moment arms (purple) provide torque advantage. See also Table S3; Figures S1–S6; Data S1.

Our qualitative analysis of EFMJ morphology and quantification of metatarsal torsion (Figure 2B, Table 2) show that despite the variation in curvature and the presence of rims and grooves affecting how the articular surfaces of the entocuneiform and the first metatarsal interlock, all specimens exhibit a saddle-shaped joint that permits combined flexion-extension (FE), abduction-adduction (ABAD), and inversion-eversion (IE) motions. The overall mobility of the EFMJ is indicated by the volume of the alpha shapes and the viable poses by the points within the pose space, including the minimum and maximum values for the combined rotation about the three rotational axes (Figures 4 and 5; Table S3; Figures S1–S6; Data S1).

**Table 2:** Morphological features of the entocuneiform and first metatarsal analyzed qualitatively or quantitatively, and their effect on hallucal grasping capabilities

Qualitative assessment	
Morphological features	Significance to hallucal grasping
Shape of the entocuneiform and metatarsal articular surfaces	categorization of joint type and function
Position of the articular surface on the entocuneiform: distal	provides the metatarsal with lower divergence
Position of the articular surface on the entocuneiform: medially tilted	provides the metatarsal with higher divergence
Presence and relative size of the peroneal process of the metatarsal	a process can expand the articular arc and/or restrict hallucal movement toward the midline of the foot through collision with the entocuneiform

**Quantitative assessment**	

Low degree of metatarsal torsion about its long axis: metatarsal’s head faces ventrally	metatarsal is flexed in ventral direction and adducted laterally toward the foot’s midline
High degree of metatarsal torsion about its long axis: metatarsal’s head is rotated toward the foot’s midline	metatarsal is moved in toward the foot’s midline with flexion and in the foot’s dorsal direction with adduction

Our osteological analysis highlighted that a saddle-shaped joint and variation in entocuneiform and metatarsal features yield greater FE potential than ABAD potential in primates and marsupials, and the converse in *T. belangeri* and rodents. The articular surface on the entocuneiform is oriented distally in primates. The peroneal process of the metatarsal is present in all primate taxa but is most conspicuous in *Microcebus*. The metatarsal’s head is rotated at least 51° toward the foot’s midline in all primates, with the highest rotation in *C. jacchus* (61° and 72°), followed by *C. pygmaea* (62°) and *Microcebus* (between 51° and 67°) (Figures 5A–5D; Figures S1–S6). This leads to FE occurring toward and away from the foot’s midline. In primates, FE motion is generally less restricted than ABAD, i.e., the joint allows larger ROM about this primary axis of movement. IE is more restricted than FE, but less than ABAD (Figure 4).

Like in primates, the articular surface of the entocuneiform is oriented distally in rodents and *T. belangeri*, which places their first metatarsal alongside the other metatarsals. These taxa have a well-developed peroneal process of the first metatarsal. The orientation of the metatarsal’s head relative to the concavity of the distal articular surface is lower than in primates and marsupials, in *T. belangeri* 14° and 20°, *M. minutus* 12° and 29°, while *T. swinhoei* barely displays metatarsal torsion with values under 2° (Figures 5E–5G). This results in the FE movement occurring in plantar and dorsal directions. In rodents and *T. belangeri*, the ABAD and IE movements are less restricted than FE, indicated by their alpha hulls elongated in the *y* and *x* axes (Figures 4 and 5E–5G).

In marsupials, the articular surface of the entocuneiform is tilted medially, hence providing the metatarsal with a higher degree of divergence from the rest of the digits. The peroneal process of the metatarsal is well developed in diprotodontians, while didelphid marsupials barely show a protuberance. The orientation of the metatarsal’s head varies among taxa: diprotodontians show the overall highest rotation (from 76° to 86°), thus higher than in primates. *Marmosa* sp., *Monodelphis domestica*, and *Gracilinanus* sp. present more intermediate values (45° and 58°, 20° and 39°, and 37°, respectively) (Figures 5H–5L). For marsupials, FE motion occurs as well toward and away from the foot’s midline, and is generally less restricted than ABAD. IE is the least restricted motion in marsupials, visible in the elongation of the alpha shape along the *x* axis (Figures 4 and 5H–5L).

Our osteological ROM analyses of the EFMJ (Figure 2C) demonstrate clear differences across taxa: primates and marsupials show greater FE than rodents and *Tupaia*, non-primates greater ABAD than primates, and marsupials the highest degree of IE of the sample. The range of FE is the largest in *Microcebus*, followed by marsupials and platyrrhines, which is visible in their *z* axis elongated alpha shapes. Rodents and *T. belangeri* display a comparatively smaller FE range. The range of ABAD is generally larger in non-primates than in primates. The range of IE is similarly small in primates and *T. belangeri*, followed by rodents, in which it is relatively larger. Marsupials have the largest potential IE. Additionally, the hallucal ROM volume in marsupials is overall larger than in primates, rodents, and *T. belangeri* (Figure 5). Marsupials display the highest alpha shape volumes, ranging from 97,460 to 271,000 cubed degrees in *M. domestica* MfN_MD01 and MfN_MD02, respectively. Primates, *T. belangeri*, and rodents pool together with values below those of the marsupials. *Microcebus* TiHo_ML01 and TiHo_MMU01 (50,600 and 47,800 cubed degrees, respectively) are followed by *M. minutus* (46,590 and 30,430 cubed degrees), *C. pygmaea* (29,800 and 20,600 cubed degrees), *M. murinus* TiHo_MMU02 (17,800 cubed degrees), *C. jacchus* ZW_CJ01 (7,450 cubed degrees), *T. swinhoei* HUB_TS02 (7,070 cubed degrees), *T. belangeri* TiHo_TB01 (6,860 cubed degrees), *M. lehilahytsara* TiHo_ML02 (6,510 cubed degrees), and *C. jacchus* DPZ_CJ02 (4,760 cubed degrees). The lowest volume is 550 cubed degrees in *T. swinhoei* HUB_TS01.

### IMMAs

**Figure 6:**
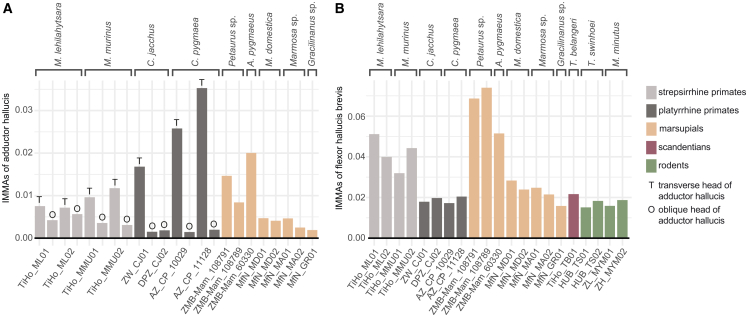
Visualization of the maximal IMMAs achieved by two intrinsic hallucal muscles (A) Adductor hallucis, where O stands for adductor hallucis oblique head and T for adductor hallucis transverse head; and (B) flexor hallucis brevis. IMMAs are normalized by muscle length and are unitless. Higher IMMAs reflect higher mechanical advantage. See also Data S1.

Our analysis of hallucal IMMAs, that estimated the moment arms of the muscles acting on the EFMJ at specific joint poses (Figure 2D), indicated that these change during the motion in a joint and differ between species (Figures 5 and 6; Data S1).

Across lineages, hallucal IMMAs show distinct clade-level patterns that transcend individual-specimen variation (Figures 5 and 6). For the adductor hallucis (transverse head), platyrrhines exhibit the largest moment arms, followed by *Microcebus*, both of which generally exceed values found in the adductor hallucis of marsupials. Within marsupials, diprotodontians show higher IMMAs than didelphimorphs, though both groups often display their strongest moment arms in flexed or moderately extended poses. Primates tend to show higher IMMAs in hallucal extension, abduction, and eversion.

For the oblique head of the adductor hallucis, strepsirrhines (*Microcebus*) exceed platyrrhines but remain below the transverse-head values of all primates and below diprotodontians, while being broadly comparable to didelphimorphs. Across primates, IMMAs tend to peak in flexed or moderately extended poses, often combined with abduction and either inversion in *Microcebus*, or eversion in platyrrhines.

The flexor hallucis brevis shows the clearest separation among clades (Figure 6): diprotodontians consistently reach the highest IMMAs, followed by didelphimorphs and *Microcebus*, while platyrrhines, rodents, and *Tupaia* achieve similar lower values. Across marsupials, larger moment arms typically occur in flexed poses (didelphimorphs) or in combinations of flexion with abduction and inversion (diprotodontians). Primates, in contrast, often show high IMMAs in flexed or extended positions combined with inversion or abduction, but with less extreme pose dependence than in arboreal marsupials. In *T. belangeri*, the muscle has larger moment arms in positions of higher abduction across the IE range and in combination with higher extension. In *T. swinhoei*, the flexor has larger moment arms in poses of higher hallucal extension, abduction, and inversion. In *M. minutus*, larger moment arms are present in poses with higher adduction.

For the flexor hallucis accessorius, didelphimorphs (e.g., *M. domestica*) exhibit substantially larger IMMAs than platyrrhines (e.g., *C. jacchus*), again with peaks in flexed poses across broad motion ranges. Finally, the contrahens I in *Tupaia* falls within the IMMA ranges of the adductors in both primates and non-primates, with larger moment arms in abducted and more extended positions (see Data S1).

Overall, small arboreal primates and marsupials, *Tupaia* and rodents differ in both magnitude and pose dependence of hallucal IMMAs, commonly achieving high moment arms in flexed poses that support grasping through combinations of flexion with inversion/eversion or abduction/adduction, suggesting different strategies for mechanical emphasis on powerful, stabilizing hallucal grasp during climbing.

## Discussion

Hallucal grasping, i.e., a specific version of pedal grasping in which the substrate is held between the hallux and the lateral digits, is achieved by various phylogenetically distant arboreal mammals, which highlights its convergent functional significance.^13^^,^^18^^,^^21^^,^^24^^,^^26^^,^^29^^,^^36^^,^^37^^,^^38^^,^^39^^,^^67^ In this study, we tested three hypotheses that focused on hallucal muscle architecture, osteological mobility, and IMMAs. We demonstrate (1) that species belonging to Primates, Scandentia, Rodentia, Diprotodontia, and Didelphimorphia that perform hallucal grasping do so differently in relation to differences in specific muscular and osteological features, and crucially, (2) that their hallucal grasping capabilities are distributed along a spectrum. In summary, our results indicate that hallucal grasping is achieved differently due to species-specific metatarsal, entocuneiform and joint morphologies, and hallux-related intrinsic muscular complexes. While all species have a saddle-shaped joint that enables the metatarsal to move in three planes combined, we can divide our dataset in two groups when it comes to the rest of the investigated features. On the one hand, small strepsirrhine and platyrrhine primates (represented by *Microcebus* and Callitrichinae, respectively) and arboreal marsupials exhibit features that make evident their convergent functional adaptations to hallucal grasping, as previously suggested.^13^^,^^23^^,^^29^^,^^31^ On the other hand, rodents and *Tupaia* share features of a less specialized hallux.^32^

### Intrinsic hallucal muscle architecture

Our comparative analysis of intrinsic hallucal musculature reveals clear architectural specializations in primates and arboreal marsupials that support elevated force-generating capacities relative to scandentians and rodents as predicted by our “muscle architecture hypothesis.” In the strepsirrhine primates *Microcebus*, both transverse and oblique heads of the adductor hallucis exhibit the largest relative volumes and ACSAs, with the oblique head, exclusive to primates, spanning the lateral sesamoid and metatarsal head to optimize moment arm leverage for combined flexion and adduction.^45^ By contrast, platyrrhine primates present smaller adductor head ACSAs, and one callitrichid, *C. jacchus* DPZ_CJ02, has reduced the complex to a single oblique head, resulting in lower predicted maximal adduction forces. Although both primate taxa share similarities, such as small body sizes and quadrupedal locomotion with leaping, foot modifications (e.g., a relatively short hallux and claw-like distal phalanges instead of flat nails) associated with the typical vertical clinging type of locomotion on large trunks of *C. jacchus* and *C. pygmaea* likely include the muscular differences compared with the other primates.^46^^,^^47^^,^^68^^,^^69^ Nevertheless, the oblique head has been proposed to play a significant role in the ability to perform powerful grasping in both *Microcebus* and callitrichids.^37^^,^^40^^,^^45^^,^^46^^,^^70^^,^^71^ Arboreal marsupials display middle-to-high ACSA variability in their adductor hallucis: all taxa possess only a single adductor hallucis head homologous to the primate transverse head, yet small genera such as *Marmosa* and *Petaurus* achieve adductor ACSAs rivaling or exceeding those of the strepsirrhine primates, suggesting convergent modification of insertion sites and cross-sectional architecture to amplify adduction and lateral rotation. The presence of the adductor hallucis in marsupials offers compelling support for the hypothesis that small arboreal marsupials exhibit parallelisms with primates in their enhanced hallucal grasping capabilities.^13^^,^^26^^,^^37^^,^^72^ Notably, the higher ACSA observed in certain diprotodontians and didelphimorphs suggests that some marsupials may be able to generate a large adducting force comparable to that produced by some primates (e.g., platyrrhines) with one or two heads of the adductor hallucis. Such previously observed convergent behavioral adaptations to arboreal locomotion have led researchers to regard some marsupials as extant analogues of ancestral primate stages.^13^^,^^23^^,^^27^^,^^35^^,^^73^ Moreover, the anatomically less differentiated adductor hallucis in marsupials may reflect an evolutionary stage akin to that of early primates. In scandentians (*Tupaia belangeri*) and rodents, however, the absence of a discrete adductor hallucis correlates with minimal transverse-head development and a generalized contrahentes-derived muscle layer, which in *T. belangeri* yields markedly lower ACSAs and, consequently, lower predicted torque around the metatarso-phalangeal joint. This may be related to a terrestrial ancestry of Tupaiinae.^32^^,^^74^ Notably, it has previously been shown that among scandentia, the arboreal genus *Ptilocercus* exhibits greater adaptation to grasping than *Tupaia*, with a greater range of hallucal abduction, functioning more similarly to primates.^18^^,^^32^^,^^46^^,^^75^ We were unable to include this rare species in this study, but future investigations of its hallucal musculoskeletal properties in a comparative context would help understanding the possible differential adaptations of this group.

Flexor hallucis brevis architecture further underscores this dichotomy. Marsupials, especially *Monodelphis*, *Marmosa*, *Gracilinanus*, and *Petaurus*, exhibit broad, latero-medial bellies often merged into dual heads, resulting in the highest brevis ACSAs among all intrinsic muscles and reflecting an insertion strategy across both sesamoids that maximizes force generating capacity for sustained flexion. *Microcebus* and platyrrhines maintain single-bellied but robust brevis muscles that, although lower in ACSA than their oblique adductors, cooperate with long flexors to maintain the grasp. Interestingly, hallucal flexion in *C. jacchus* depends only on this muscle since the long flexors do not supply this digit. In stark contrast, *T. belangeri* and rodents confine their intrinsic hallux flexion to a muscle with low ACSA, indicating modest flexion capabilities consistent with their generalized grasping strategies. It is noteworthy, however, that the rodent *Micromys minutus* has been documented to aptly climb thin vertical supports^25^^,^^28^^,^^76^ and appears to rely on the flexor hallucis brevis and specialized tendon-locking mechanisms to balance power and energy economy during prolonged grips.^41^

Accessory muscles exhibit patchy distributions reflecting species-specific hallucal grasping strategies: flexor hallucis accessorius appears only in *Callithrix jacchus* and *Monodelphis domestica*, suggesting either retention of an ancestral pattern or independent gain. The low and variable ACSAs of this muscle imply an auxiliary role. Uniquely, *T. belangeri* expresses a plantar bony plaque and a contrahens I muscle, yet its overall ACSA profile remains low. Interestingly, it has been suggested that the adductor hallucis (discussed above) is derived from the contrahentes muscle layer.^40^^,^^77^

Collectively, these architectural patterns, variation in muscle head number, ACSA, and specialized insertion sites, map directly onto each clade’s arboreal niche and corroborate our hypothesis that primates and arboreal marsupials possess enhanced intrinsic muscle design features that allow higher force-generating capacities than those of scandentians and rodents, thereby underpinning their greater abilities for more powerful grasping. Nevertheless, our results highlight the presence of a spectrum of “powerfulness” in grasping capabilities, realized by diverse anatomical conditions, instead of the previously suggested dichotomy between powerful and non-powerful, the former largely attributed to primates.

### Hallucal osteology and joint mobility

Our results largely support our “osteological mobility hypothesis,” which predicts that primates and arboreal marsupials exhibit enhanced three-dimensional ROM at the entocuneiform-first metatarsal joint (EFMJ) compared to scandentians (*Tupaia*) and rodents, reflecting their convergent specializations for hallucal grasping. All taxa share a saddle-shaped EFMJ permitting flexion-extension (FE), abduction-adduction (ABAD), and inversion-eversion (IE) yet clear osteological and kinematic morphotypes emerge. In primates and marsupials FE consistently exceeds ABAD, and between taxa, strepsirrhines (*Microcebus*) show the generally largest FE excursions, followed by marsupials, then platyrrhines. *Tupaia* and rodents reverse this pattern, exhibiting less constrained ABAD and IE but restricted FE and greater ABAD than primates. These motion hierarchies mirror facet orientation and metatarsal torsion: primates in this dataset possess a distally facing entocuneiform and a pronounced peroneal process that expands the articular arc (especially in *Microcebus*), combined with 51°–72° lateral torsion of the metatarsal head to drive hallux divergence and power flexion-adduction synergy. The rotation of the metatarsal’s head toward the foot’s midline increases hallucal contact with the surface during grasping, through simultaneous adduction, flexion, and inversion of the metatarsal.^46^^,^^70^ Instead, in marsupials, the entocuneiform facet is tilted medially, achieving a permanently divergent hallux. Diprotodontians show extreme head torsions (76°–86°) and consistently well-developed peroneal processes, whereas didelphimorphs display more moderate torsion values (20°–58°) and inconspicuous peroneal process morphologies. We thus confirm that the diverging first digit is the most evident convergent adaptation for grasping between marsupials and primates.^7^^,^^16^^,^^23^^,^^26^^,^^29^^,^^36^^,^^37^^,^^38^^,^^71^^,^^72^^,^^73^^,^^78^^,^^79^ This shared feature, even though achieved by different anatomical conditions, together with the epiphyseal torsion results in an advantageous hallucal position where forces can be more effectively applied by the ventral surface of the hallux on a curved support like a branch^80^ underlining the convergence between these two lineages. By contrast, *Tupaia* and rodents exhibit minimal torsion (<30°) and distal facets that align the hallux parallel to the lateral digits (see Sargis^32^ for detailed foot morphology in *Tupaia*), as previously observed in their pedal postures,^13^ and structurally favoring ABAD-driven grasp.^32^^,^^74^ Indeed, previous detailed analyses of the pedal grasping postures have found that in contrast to primates and marsupials, in *Tupaia* and rodents it is the lateral surface of the hallux that is in contact with the support during a grasp.^13^

Absolute cosine-corrected ROM volumes, computed as the product of FE, ABAD, and IE excursions, are highest in marsupials, intermediate in *Microcebus* and rodents, and lowest in platyrrhines, *Tupaia*, and one specimen of *T. swinhoei*. The conspicuously large osteological ROM found in marsupials is likely conferred by a broad range of potential inversion-eversion. Although primate ROM volumes can overlap those of rodents and *Tupaia*, which challenges our hypothesis, the pattern of FE dominance in primates and marsupials versus ABAD dominance in non-primates underscores functional partitioning: primates and marsupials leverage combined FE-ABAD movements, unified by lateral torsion of their metatarsal heads, to apply force efficiently on curved arboreal supports, whereas rodents and *Tupaia* rely on a more generalized, ABAD-centric grasp. Morphological trade-offs further refine this spectrum even though it remains unknown to what extent the motion is retained in each species when soft tissue comes into play, e.g., ligaments.^71^ Large peroneal processes in primates bolster joint stability at the expense of maximal flexion, while in *Tupaia* the thick aponeurotic band binding the first metatarsal to the plantar fascia and a reportedly restricted EFMJ constrains FE to its observed lower bounds, the metatarso-phalangeal joint being likely primary source of hallucal movement.^32^^,^^46^^,^^74^ In marsupials, soft tissues likely constrain IE far beyond the effects of osteological morphology that are captured in our ROM analyses. A joint’s osteological ROM can be additionally bound to more articulation constraints^81^ or increased by translational degrees of freedom.^82^ Our approach is, however, a solid evaluation of joint three-dimensional mobility that provides biologically meaningful information about general differences among the taxa, and that together with joint architecture, further corroborates a spectrum of hallucal grasping specializations that align with each clade’s locomotor behavior and the hypothesized link between enhanced FE/ABAD capacity and powerful hallux-mediated grasp.

### Hallucal IMMAs

With respect to our “moment-arm hypothesis,” our comparative analysis of instantaneous muscular moment arms (IMMAs) across primates, arboreal marsupials, scandentians, and rodents reveals biomechanical refinements in primates and diprotodontian and didelphimorph marsupials that enhance hallucal-grasp torque. IMMAs have been documented to reflect ecomorphological specialization in closely related taxa^61^ as well as larger-scale evolutionary transitions.^60^ In this study, all taxa exhibit pose-dependent moment arm shifts at the entocuneiform-metatarsal joint and achieve high IMMAs in key hallucal muscles under specific joint configurations, which underlines the differences in grasping strategies. While the hypothesis of full convergence between primates and marsupials in IMMA-based force optimization is not fully supported, the results reveal partial functional convergence: primates outperform marsupials in adductor hallucis torque generation, whereas diprotodontians surpass all other groups in flexor hallucis brevis performance. Furthermore, our results indicate that combinations of FE-ABAD-IE increase IMMAs more than single-plane movements across all lineages and for all muscles, suggesting multi-planar optimization strategies. Together, these patterns form a continuum of moment-arm capacities across mammals, reinforcing the view that autopodial grasping and arboreal adaptation represent a spectrum rather than discrete categories.

These findings further suggest that *in vivo* grasping strength—as emerging from studies such as Granatosky et al.^33^—is likely to reflect this pattern of partial convergence, with primates and arboreal marsupials producing higher grasping forces, scandentians and rodents possibly producing intermediate forces, and other less specialized arboreal models producing the weakest forces. Importantly, achieving a complete understanding of hallucal grasping also requires investigation of the osteological and muscular mechanisms of the lateral digits, as well as their interplay with the extrinsic foot muscles.

Several species or genera investigated in this work have previously been studied in the context of functional significance of grasping in arboreal locomotion (see Nyakatura^35^ for a review and Toussaint et al.^13^). *Microcebus*, callitrichids, tupaiids, and marsupials have been used as extant models to represent and interpret different stages of evolution of early primates and their grasping capabilities.^18^^,^^23^^,^^35^^,^^83^ A refined understanding of pedal grasping adaptations in extant taxa informs fossil interpretations, as taxa such as plesiadapiforms, for example, exhibit diverse pedal specializations consistent with arboreal locomotion, an insight that is crucial for reconstructing early primate evolutionary pathways (e.g., Chester et al.^84^).

We revisited the notion of powerful hallucal grasping in small arboreal mammals, long held as a key innovation in primate origins, through an integrative analysis of muscle properties, joint mobility, and lever-arm mechanics. Rather than a strict powerful versus non-powerful divide, our results reveal a functional continuum in hallucal grasping adaptations. All taxa share a saddle-shaped entocuneiform—first metatarsal joint permitting flexion-extension (FE), abduction-adduction (ABAD), and inversion-eversion (IE). Primates and marsupials bias this mobility toward flexion-extension, displaying different feature combinations of pronounced metatarsal’s facet torsion, enlarged peroneal processes, and a tilted entocuneiform articular surface. In contrast, scandentians and rodents emphasize abduction-adduction with minimal facet torsion and no articular surface tilt at the entocuneiform. Interestingly, marsupials outperform primates in overall osteological ROM at this joint, highlighting the convergent specialization of their grasping capabilities for locomotion on arboreal supports. While extrinsic hallucal musculature adds an additional layer of complexity to the functional morphology of hallucal grasping, our analysis of intrinsic hallucal musculature reveals a spectrum of strategies and nuanced differences in the effectiveness of hallucal grasping within small arboreal mammals and even between primates and arboreal marsupials. Some primates possess larger cross-sectional adductor hallucis, while marsupials have larger cross-sectional flexor hallucis brevis muscles, essential for sustained torque. *Tupaia* and rodents maintain simpler, lower cross-sectional muscle complexes, likely relying more on extrinsic muscles and passive tissues. Moment-arm mechanics reinforce the continuum. All lineages achieve peak IMMAs in key internal hallucal muscles under specific joint poses, some but not all emphasizing high-flexion and high-adduction torque. Coupling rotations consistently elevates IMMAs, underscoring a multi-planar optimization for torque. Overall, we found osteological and myological refinements in each clade that align with generalized arboreal locomotion such as vertical climbing and horizontal walking. Importantly, our study reframes powerful hallucal grasping as a spectrum of morphofunctional solutions reflecting that morphological adaptations to hallucal grasping were likely acquired independently during mammalian evolution to arboreal life. Future work integrating fiber-type histochemistry, *in vivo* electromyography, 3D musculoskeletal modeling of extrinsic grasping muscles, and measurements of *in vivo* grasping forces across species will further illuminate the dynamic performance and evolutionary trajectories of these diverse grasping strategies.

### Limitations of the study

Several aspects of this study should be considered when interpreting the results. Although the comparative design of this investigation offered valuable insights into consistent patterns across individuals of 13 species, the limited number of available individuals per species prevented the use of formal statistical analyses. Moreover, muscle fiber tracking and the calculation of the physiological cross-sectional area (PCSA) as a likely more direct indicator of a muscle’s capacity to generate force would provide more refined estimates of muscular functional differences (but see Lieber^85^). Investigating muscular architecture and moment arms of extrinsic grasping muscles and muscular and osteological mechanism of the lateral digits in addition to what was achieved here would offer a more complete understanding of an individuals’ grasping capabilities, leaving room for future analyses. Using a six-degree-of-freedom framework for evaluating the joint’s osteological ROM, i.e., allowing translations within a joint in addition to the combined rotations analyzed in the current study, can be expected to more accurately capture the complexity of its mobility.^82^^,^^86^

## Resource availability

### Lead contact

Requests for further information and resources should be directed to and will be fulfilled by the lead contact, Irene Montañez-Rivera (irene.montanezrivera@uantwerpen.be).

### Materials availability

This study did not generate new materials.

### Data and code availability

All data reported in this paper will be shared by the lead contact upon request. This paper does not report original code. Any additional information required to reanalyze the data reported in this paper is available from the lead contact upon reasonable request.

## Acknowledgments

The authors would like to thank Franziska Eberhardt and Luisa Merten for their assistance in data processing; Heiko Stark for helpful advice; and Adrian Scheidt and the members of the Nyakatura Lab for insightful discussions in regards of *grasping grasping*. We thank directors, curators, and managers from Antwerp Zoo (Belgium) and Deutsches Primatenzentrum (Germany) for providing specimens, as well as Peter Giere and Detlef Willborn from Museum für Naturkunde Berlin (Germany), Karoline Albig from Zoo Halle (Germany), Christina Schubert from Zoo Landau (Germany), and Pierre Grothmann from Zoo in der Wingst (Germany). The photography of *M. murinus* is attributed to Marina Scheumann. A.R.M. was supported by the US National Science Foundation Postdoctoral Research Fellowship in Biology (PRFB (DBI-2209144)) and Yale Institute for Biospheric Studies Gaylord Donnelley Postdoctoral Environmental Fellowship. S.L.D.T. was supported by an Alexander von Humboldt Foundation postdoctoral fellowship (1210538-FRA-HFST-P). J.A.N. was supported by the 10.13039/501100001659German Research Foundation (NY 63 2/1).

## Author contributions

I.M.R., conceptualization (lead), data curation (lead), formal analysis (lead), investigation (lead), methodology (lead), project administration (supporting), software (lead), supervision (supporting), visualization (lead), writing – original draft (equal), writing – review and editing (equal); S.L.D.T., conceptualization (supporting), data curation (supporting), formal analysis (supporting), funding acquisition (supporting), investigation (lead), methodology (supporting), project administration (supporting), software (supporting), supervision (supporting), visualization (lead), writing – original draft (equal), writing – review and editing (equal); A.S., data curation (supporting), resources (supporting), writing – review and editing (equal); A.R.M., data curation (supporting), formal analysis (supporting), methodology (supporting), software (supporting), writing – review and editing (equal); U.R., resources (supporting), writing – review and editing (equal); V.B., formal analysis (supporting), investigation (supporting), visualization (supporting), writing – review and editing (equal); M.N., formal analysis (supporting), investigation (supporting), visualization (supporting), writing – review and editing (equal); J.A.N., conceptualization (lead), funding acquisition (lead), investigation (supporting), methodology (lead), project administration (lead), resources (lead), supervision (lead), visualization (supporting), writing – original draft (equal), writing – review and editing (equal).

## Declaration of interests

The authors declare no competing interests.

## STAR★Methods

### Key resources table

**Table:** 

REAGENT or RESOURCE	SOURCE	IDENTIFIER
**Experimental models: Organisms/strains**		

*Microcebus lehilahytsara*	Stiftung Tierärztliche Hochschule Hannover, Germany	Ute Radespiel
*Microcebus lehilahytsara*	Stiftung Tierärztliche Hochschule Hannover, Germany	Ute Radespiel
*Microcebus murinus*	Stiftung Tierärztliche Hochschule Hannover, Germany	Ute Radespiel
*Microcebus murinus*	Stiftung Tierärztliche Hochschule Hannover, Germany	Ute Radespiel
*Callithrix jacchus*	Zoo in der Wingst, Germany	Pierre Grothmann
*Callithrix jacchus*	Deutsches Primatenzentrum, Göttingen, Germany	John A. Nyakatura
*Cebuella pygmaea*	Antwerp Zoo (Royal Zoological Society of Antwerp), Belgium	John A. Nyakatura
*Cebuella pygmaea*	Antwerp Zoo (Royal Zoological Society of Antwerp), Belgium	John A. Nyakatura
*Tupaia belangeri*	Stiftung Tierärztliche Hochschule Hannover, Germany	Ute Radespiel
*Tupaia belangeri*	Stiftung Tierärztliche Hochschule Hannover, Germany	Ute Radespiel
*Tamiops swinhoei*	Humboldt-Universität zu Berlin, Germany	John A. Nyakatura
*Tamiops swinhoei*	Humboldt-Universität zu Berlin, Germany	John A. Nyakatura
*Micromys minutus*	Zoo Landau, Germany	Christina Schubert
*Micromys minutus*	Zoo Halle, Germany	Karoline Albig
*Petaurus* sp.	Museum für Naturkunde Berlin, Germany	Detlef Willborn
*Petaurus breviceps*	Museum für Naturkunde Berlin, Germany	Detlef Willborn
*Acrobates pygmaeus*	Museum für Naturkunde Berlin, Germany	Detlef Willborn
*Monodelphis domestica*	Museum für Naturkunde Berlin, Germany	Peter Giere
*Monodelphis domestica*	Museum für Naturkunde Berlin, Germany	Peter Giere
*Marmosa* sp.	Museum für Naturkunde Berlin, Germany	Detlef Willborn
*Marmosa* sp.	Museum für Naturkunde Berlin, Germany	Detlef Willborn
*Gracilinanus* sp.	Museum für Naturkunde Berlin, Germany	Detlef Willborn

**Software and algorithms**		

Amira 6.0.0	Thermo Fisher Scientific, Waltham, MA, U.S.A.	https://www.thermofisher.com/be/en/home/electron-microscopy/products/software-em-3d-vis/amira-software.html?SID=srch-srp-AMIRA
Amira ZIB edition 2021.03	Thermo Fisher Scientific, Waltham, MA, U.S.A. & Konrad-Zuse-Zentrum für Informationstechnik Berlin (ZIB), Germany	https://www.thermofisher.com/be/en/home/electron-microscopy/products/software-em-3d-vis/amira-software.html?SID=srch-srp-AMIRA
Geomagic Studio 2013	Geomagic, Inc., Research Triangle Park, NC, U.S.A.	https://hexagon.com/products/geomagic-wrap
Autodesk Maya 2020	Autodesk, Inc., San Rafael, CA, USA	https://www.autodesk.com/products/maya/overview
R version 2024.04.2	The R Foundation, Vienna, Austria	https://www.r-project.org/
Plotly	Plotly Technologies Inc., London, UK	https://plotly.com/r/

### Experimental model and study participant details

#### Specimen collection and diceCT

Anatomical and biomechanical data were collected from a total of 22 individuals of 13 mammalian species (Table 1), selected according to the following criteria: a) known display of arboreal and scansorial locomotion, b) body mass lower than 500 g, c) adult stage. Frozen specimens, or specimens preserved in alcohol were acquired from various collaborators (Tables 1 and S4). No animals were sacrificed for this study. All cadavers originated from animals that had died naturally in zoological gardens and laboratory animal housing facilities. Body masses were provided by the institutions of origin or were measured directly for each complete specimen using a standard weighing scale. As the organs of the *Petaurus breviceps* specimen (ZMB-Mam_108789) had been previously extracted from the body, we used the average body mass of 97.5g available in the literature.^87^ Information on sex and age was provided by the institutions of origin (Table 1). The ontogenetic stage remains unknown for all diprotodontians but adulthood is assumed based on epiphyseal fusion. The limited number of available individuals prevented reporting the influence of sex on the results.

### Method details

The right foot was detached at the distal half of the tibia and fibula. It was then skinned and kept moist with water during dissection to avoid muscle desiccation. The skinned right foot of all specimens was subjected to contrast-enhancing staining (methodology modified from Metscher^88^^,^^89^). Specimens were fixed overnight in 4.1% formalin and 1x phosphate-buffered saline, then transferred to a water bath, followed by dehydration via ascending ethanol concentrations (15% for 0.5 h, and 30%, 50%, 60%, and 70% for 1 h each). Subsequently, each specimen was stained in 100 mL of 1% iodine dissolved in 70% ethanol under gentle agitation. Staining durations varied from 2 to 7 days depending on sample volume (see Table S4).

We performed high-resolution μCT scans of the right feet with an isometric voxel size ranging from 2.6 to 11.0 μm (Tables 1 and S4). From the acquired high-resolution image stacks, individual bones and hallux-related intrinsic muscles were segmented with the software Amira and Amira ZIB edition (6.0.0 and 2021.03 respectively, Thermo Fisher Scientific, Waltham, MA, U.S.A.). Subsequently, 3D surface models of each segmented muscle and bone were generated, each comprising 30.000 polygons to reduce the computational load. The models were refined and visualized using Geomagic Studio 2013 (Geomagic, Inc., Research Triangle Park, NC, U.S.A.) and Autodesk Maya 2020 (Autodesk, Inc., San Rafael, CA, USA) (Figure 2A).

### Quantification and statistical analysis

#### Muscle topology

During segmentation, the position, orientation, origin, and attachment sites of the hallux-related intrinsic muscles were documented for myological descriptions. Anatomical comparisons were based on existing descriptions of several taxa, and literature on closely related species was used for those lacking prior myological descriptions: Beattie,^90^ Casteleyn & Bakker,^91^ and Langdon^92^ for *C. jacchus*; Beattie^90^ and Hill^93^ for *C. pygmaea*; Hafferl,^94^ Gebo^71^ and Langdon^92^ for *Microcebus*; Hoffmann,^95^ Parsons,^96^ Peterka,^97^ Orwoll^98^ and Bryant^99^ for *T. swinhoei*; Parsons^96^ and Greene^100^ for *M. minutus*; Carlsson,^101^ Le Gros Clark,^102^^,^^103^ Verma,^104^ George^105^ for *T. belangeri*; Stein^106^ and Coues^107^ for didelphimorphs. No previous descriptions were found for diprotodontians.

#### Muscle architecture

We investigated the muscular architectural parameters of volume, length and ACSA of the intrinsic muscles involved in hallucal adduction and flexion, namely, adductor hallucis, flexor hallucis brevis, flexor hallucis brevis accessorius and contrahens I (Figure 2A)*.* On a general level, volume reflects the muscle’s potential force production, since muscles larger in volume generally have a larger number of fascicles arranged in parallel.^108^^,^^109^^,^^110^ To limit metabolic costs, the volume is expectedly minimized as long as the functional and biological roles are not compromised. Since the ability of muscles to generate force depends on their cross-sectional area, we used the ACSA of a muscle as an alternative indicator of force generation capacity. ACSA, in contrast to the physiological cross-sectional area, does not account for the pennation angle of the fibers, which was argued to likely serve only as a fiber packaging strategy.^85^ The larger the ACSA, the more likely a muscle is adapted to movements that require great force, in this case, for more powerful grasping.

#### Muscle volume and length

After segmentation, each muscle’s volume was extracted automatically from the CT scans using the function ‘material statistics’ in Amira. To account for storage effects, volume corrections were applied as follows: multiplication by a factor of 1.64 for AZ_CP_M11128, AZ_CP_M10029, and HUB_TS01 due to short-term ethanol storage^111^; by a factor of 2.49 for specimens ZMB-Mam_108791, ZMB-Mam_108789, and ZMB-Mam_60330 due to long-term ethanol storage^112^; and by a factor of 1.32 for AZ_CP_M11128 and AZ_CP_M10029 due to over one month of exposure to 4.1% formalin.^111^ All corrected volumes were used in subsequent analysis (see Figure S7).

The length of muscles was measured on the generated muscle surfaces using Geomagic Studio 2013. The center of the proximal and distal parts of each muscle where it is associated with tendons or attached to bones was determined (“Features”, “Point” and “Center” tools) and the distance between these two points was automatically measured in mm. For curved muscles, a landmark was placed in the middle and the two measures were added. For muscles with more than one belly, the mean length was calculated.

#### ACSA

The ACSA of each muscle was calculated as ACSA = TotVolume/TotLength. To account for size differences, the Pearson correlation coefficient in R version 2024.04.2 was used to test for linear correlation between body mass and volume, length, and ACSA in all muscles that were present in more than two species. If correlated, values for volume, length, and ACSA were corrected with body mass, assuming isometric scaling as follows: Vol_rel_ = volume/body mass, and Length_rel_ = length/body mass^0.33^, and ACSA_rel_ = ACSA/body mass^0.66^ (see Figure S7).

#### Joint morphology and metatarsal torsion

We qualitatively characterized several aspects of the entocuneiform-first metatarsal joint (EFMJ) morphology that may influence the adduction and flexion of the hallux (Figure 2B, see Figures S1, S2, S3, S4, S5, and S6 for skeletal models). These aspects are listed in Table 2 along with an explanation of how each may affect hallucal movement. Additionally, we quantified the degree of torsion of the metatarsal about its long axis, since the orientation of the metatarsal’s head bearing the muscles’ attachment sites influence the direction of the flexing and adducting movements (Table 2).

To measure the metatarsal’s torsion, a cylinder was placed fitting the curvature of the metatarsal’s head, and its long axis was oriented parallel to the sesamoid bones. A second cylinder was fitted in the concave part of the distal articular surface of the metatarsal that enables the movement toward and away from the foot’s midline. The area of the proximal and distal articular surfaces of the metatarsal was automatically calculated; a coordinate system was placed at the center of each area and oriented so that their x axes overlap. The z axis of the distal coordinate system in rodents and *T. belangeri* was oriented parallel to the long axis of the distal cylinder, while the y axis of the proximal coordinate system was parallel to the long axis of the proximal cylinder. In primates and marsupials, the y axis of the distal coordinate system was parallel to the long axis of the distal cylinder, while the z axis of the proximal coordinate system was parallel to the long axis of the proximal cylinder. In all specimens, the metatarsal’s torsion was measured as the difference in degrees of rotation by the x axis between both coordinate systems (angle α).

#### Osteological range of motion (ROM)

We quantified mobility at the entocuneiform-fist metatarsal joint (EFMJ) for each specimen using an automated assessment of the osteological ROM. Osteological ROM does not consider limitations imposed by soft tissue as ligaments, muscles, and tendons, but yields an insight into the functional significance of joint osteological morphology. We focus only on the EFMJ for its pivotal role in enabling flexion-extension (FE) and crucially, the abduction-adduction (ABAD) movement of the hallux, thus considering the movement of the first metatarsal relative to the entocuneiform and disregarding the roles of other foot bones. Previous studies on ROM often concentrated on the minimum and maximum motion of a joint’s rotational degree of freedom and considered only uniaxial movements.^113^^,^^114^^,^^115^^,^^116^ Here, we model more biologically meaningful 3D ROM with rotational degree of freedom interactions^117^^,^^118^^,^^119^ to evaluate species’ differences in osteological specialization to hallucal grasping.

Following Manafzadeh & Padian^118^ we determined osteological ROM from the 3D joint models in Autodesk Maya based on Maya embedded language (MEL) scripts,^118^^,^^120^ which were here extended to accommodate specific constraints of the hallucal movement. First, a sphere was placed between entocuneiform and metatarsal I to define the interarticular space. A forward kinematic rig was created with the entocuneiform, a joint coordinate system (JCS) and metatarsal I in hierarchical sequence. The JCS was placed at the center of rotation (COR) of the EFMJ of each specimen, the position of which was approximated by fitting a second sphere between entocuneiform and metatarsal I, matching the surface of this sphere with the curvature of the metatarsal’s articular surface (Figure 2C). The axes of the JCS were aligned relative to the articular surface of the entocuneiform. The x axis was always oriented perpendicular to the surface. In primates and marsupials the y axis was oriented along the medio-lateral plane, thus rotation about this axis moved the metatarsal toward the dorsal plane (adduction) or toward the ventral plane of the foot (abduction), while the z axis was oriented along the dorsoventral plane of the articular surface, hence rotation about this axis moved the metatarsal toward (flexion) and away from the foot’s midline (extension; Figure 2C). In rodents and tupaiids the y axis was oriented along the dorsoventral plane of the entocuneiform’s articular surface, resulting in the first metatarsal moving toward (adduction) and away from the foot’s midline (abduction), while the z axis was placed along the medio-lateral plane of the articular surface, thus rotation about this axis moved the metatarsal toward the ventral plane of the foot during flexion and toward the dorsal plane during extension. In this manner, movement around the COR rotated metatarsal I relative to the entocuneiform such that rotation about the z axis corresponded to FE, while rotation about the y axis corresponded to ABAD, and x axis to inversion-eversion (IE) in all specimens. The reference pose of the joint (i.e., with the coordinates 0/0/0 in the JCS) was established for all specimens as follows: the x axis of the metatarsal’s proximal coordinate system created for measurement of the metatarsal torsion was placed overlapping the x axis of the JCS, and their respective y- and z-axes were placed parallel to each other and oriented to the same direction. During alignment into the reference pose, the spacing defined by the first sphere was preserved.

In this automated assessment of the osteological ROM we considered combinations of all 3° of freedom (DOF) simultaneously but set reasonable limits to avoid presumed unrealistic movements (e.g., the rotation of the metatarsal by over 90° around its long axis). The ranges were 270° for FE and 180° for ABAD and IE and encompassed the rotation limits for all EFMJ about each axis that were determined by collision of metatarsal and entocuneiform bone surfaces. Hypothetically possible joint positions were automatically calculated using the Tait-Bryan angles convention in *z-y-x* order with a fixed step size of 5°. In this way, rotation about the *z* axis also moved the *y-* and *x* axes as well as the bone model; rotation of the *y* axis also rotated the *x* axis and the model; rotation of the x axis only moved the model. To differentiate viable poses (coded “1”, no intersection mesh created) from non-viable poses (coded “0”, intersection mesh created) during rotation, the Boolean intersection of the metatarsal and entocuneiform bone meshes indicating interpenetration was calculated. All reference poses were viable. All viable poses were visually inspected and poses were categorized disarticulated when the metatarsal articular surface no longer faced the entocuneiform’s articular surface. Based on this visual determination of articulation, viable-coded poses with angles of rotation in positive z direction above certain values (60°, 20°, 50°, 10°, 70°, and 15°) were removed in the specimens MfN_GR02 (*Gracilinanus* sp.), MfN_MD01, MfN_MD02 (*M. domestica*), MfN_MA01, MfN_MA02 (*Marmosa* sp.), and ZH_MYM02 (*M. minutus*), respectively, and below −55° in ZL_MYM01 (*M. minutus*).

A cosine correction of the 3D pose space was then conducted for each joint to resolve the inherent distortion of Euler angle space and enable quantitative comparison.^120^ The alpha shapes for the pose spaces were created in MATLAB (Version R2016b), setting an alpha radius (i.e., the degree of “tightness” of the convex hull around the data points) of 50, the smallest critical value encompassing the majority of the automatically determined critical values of the dataset. If a critical value surpassed 50, this was used instead (Table S3). The alpha shapes and viable poses for all specimens were plotted on a 3D graph using the software R and the package “plotly”.^121^ The minimal and maximal values for all three DOF were plotted for a better overview.

The volume of the alpha shape indicates the size of the overall region of joint pose space occupied by the viable poses of the first metatarsal respective to the entocuneiform during combined motion about the three rotational axes and is a comprehensive metric to characterise the range of motion of the joint. The volume of the alpha shapes in cubed degrees resulting from the number of poses, and the 3D pose space occupation of the EFMJ of 21 specimens (*T. belangeri* TiHo_TB02 was excluded for exhibiting only two viable poses) was used for comparison between taxa.

#### Instantaneous muscle moment arms (IMMAs)

The functional modeling approach implemented here was conducted using Autodesk Maya 2020 with a similar methodology to Löffler et al.^61^ IMMAs at the EFMJ were estimated using for each specimen and each muscle the same foot model, forward kinematic rig, COR and calculated viable poses from the previous osteological ROM analysis (Figure 2D). For every muscle, a “locator” was positioned at each origin and insertion. The straight line extending through both locators, a simplified representation of the muscle’s line of action MLOA, contained a mobile third locator. While the EFMJ model replicated each calculated viable pose, the third locator slid on the extended MLOA, projecting the nearest point between the line of action and the COR of the EFMJ contained in the kinematic rig. The nearest point could either fall on the MLOA or outside of it, depending on the pose. The instantaneous muscle moment arm (IMMA) length for each pose was obtained from calculating the distance between the nearest point and the COR. To convert IMMA measurements from Maya units to the metric system, the ratio of the length between the calcaneal tuberosity and the fifth metatarso-phalangeal joint of the specimens (a) and the skeletal model (b) was used as a scaling factor: IMMA (mm) = a (mm)/b (Maya units) ∗ IMMA (Maya units). These foot landmarks were used as they are not covered by pads, thus allowing a consistent linear measurement in all specimens. The resulting values were normalized by muscle length for comparison, are thus unitless, and were plotted by color on the data points of the specimens’ alpha shapes, i.e., the viable poses in EFMJ motion. For visualization of the magnitude of a muscle’s IMMA and its distribution in the 3D pose space, the color scale was based on each muscle’s IMMA range (for plots with a unified scale representing the full IMMA range of a muscle across all specimens, see Data S1).

## Supplemental information

Document S1. Figures S1–S7 and Tables S3, and S4

Table S1. Muscle topology Anatomical description of the hallux-related intrinsic muscles of all specimens reporting muscle origin, course, and insertion site. This table is related to Figure 3

Table S2. Muscle architecture Architectural parameters of the hallux-related intrinsic muscles of all specimens, including volume (Vol), length (Length), and anatomical cross-sectional (ACSA) are reported as absolute values. Volume, length, and ACSA are also reported relative to body mass (rel). Values for volume and ACSA are also corrected for ethanol storage (cf) and corrected for ethanol storage and body mass (rel_cf). This table is related to Figure 3

Data S1. Joint range of motion (cosine-corrected, cc) with color-coded instantaneous muscle moment arms of hallux-related intrinsic muscles Interactive plots of the overall mobility of the EFMJ of each specimen represented by an alpha shape in a 3D pose space. The *x*, *y*, and *z* axes represent inversion-eversion (IE), abduction-adduction (ABAD), and flexion-extension (FE), respectively. Angles in degrees. The instantaneous muscle moment arm (IMMA, unitless) calculated for each muscle at each pose is color-coded in a unified scale representing the full IMMA range of a muscle across all specimens. Poses with higher moment arms (darker colors) provide torque advantage. Muscles: flexor hallucis brevis, flexor hallucis accessorius, adductor hallucis, adductor hallucis oblique head, adductor hallucis transverse head, and contrahens I. Adductor hallucis and adductor hallucis transverse head are pooled in the same color scale. This material is related to Figures 5 and 6; Table S3
