# Supplementary material for: Powerful or non-powerful? Revisiting hallucal grasping as a key evolutionary innovation in small arboreal mammals
Source: iScience. 2026 Apr 29;29(6):115913. doi: 10.1016/j.isci.2026.115913 (PMC13196134; doi:10.1016/j.isci.2026.115913)

iScience, Volume *29*

Supplemental information

Powerful or non-powerful? Revisiting hallucal grasping as a key evolutionary innovation in small arboreal mammals

Irene Montañez-Rivera, Séverine L.D. Toussaint, Alexander Stoessel, Armita R. Manafzadeh, Ute Radespiel, Vera Bruhn, Marie Newbon, and John A. Nyakatura

Document S1

Table S3
Table S4
Figure S1
Figure S2
Figure S3
Figure S4
Figure S5
Figure S6
Figure S7

Table S3. Osteological range of motion

Osteological range of motion analysis at the entocuneiform-first metatarsal joint (EFMJ) yielded a specific number of valid poses for each specimen (number_poses). The alpha and critical values of the resulting alpha shapes and their total volumes in cubed degrees are reported here. This table is related to Figure 5.

Species	Order	Specimen	total_vol_cubed_deg	alpha_value	critical_value	number_poses
Gracilinanus sp.	Didelphimorphia	MfN_GR01	246000	50	8.4101	2180
M. domestica	Didelphimorphia	MfN_MD01	97462	56.5206	56.5206	924
M. domestica	Didelphimorphia	MfN_MD02	271130	50	17.807	2325
Marmosa sp.	Didelphimorphia	MfN_MA01	131780	50	7.8463	1294
Marmosa sp.	Didelphimorphia	MfN_MA02	185210	102.3518	102.3518	1017
A. pygmaeus	Diprotodontia	ZMB-Mam_60330	105220	50	17.6241	988
P. breviceps	Diprotodontia	ZMB-Mam_108789	229254	50	14.2966483	2122
Petaurus sp.	Diprotodontia	ZMB-Mam_108791	118900	50	15.5718	1134
C. jacchus	Primates	DPZ_CJ02	4760	50	22.6538	69
C. jacchus	Primates	ZW_CJ01	7449	50	8.2887	88
C. pygmaea	Primates	AZ_CP_M10029	20600	50	8.5539	229
C. pygmaea	Primates	AZ_CP_M11128	29800	50	8.3336	301
M. lehilahytsara	Primates	TiHo_ML02	6506	50	15.9361	85
M. lehilahytsara	Primates	TiHo_ML01	50615	50	4.4151	507
M. murinus	Primates	TiHo_MMU02	17766	50	8.2887	197
M. murinus	Primates	TiHo_MMU01	47834	50	8.2887	500
M. minutus	Rodentia	ZL_MYM01	30427	50	9.0313	359
M. minutus	Rodentia	ZH_MYM02	46585	50	20.1519	404
T. swinhoei	Rodentia	HUB_TS01	550.704	50	43.8277	20
T. swinhoei	Rodentia	HUB_TS02	7071	50	8.2657	92
T. belangeri	Scandentia	TiHo_TB01	6857	50	25.1922	104
T. belangeri	Scandentia	TiHo_TB02	n.a.	n.a.	n.a.	n.a.

Table S4. Specimen collection and diceCT
A total of 22 individuals of 13 mammalian species were collected. Information on acquisition, storage and scanning is reported here. This table is related to STAR Methods.

Species	Specimen	Age	Sex	Body part	State	Storage prior fixation	Storage period	Fixative
Microcebus lehilahytsara	TiHo_ML01	8y 1m	m	right foot	deep frozen	deep frozen	NA	4.1% formalin
Microcebus lehilahytsara	TiHo_ML02	4y 1m	m	right foot	deep frozen	deep frozen	NA	4.1% formalin
Microcebus murinus	TiHo_MMU01	6y 6m	m	right foot	deep frozen	deep frozen	NA	4.1% formalin
Microcebus murinus	TiHo_MMU02	8y 9m	f	right foot	deep frozen	deep frozen	NA	4.1% formalin
Callithrix jacchus	ZW_CJ01	10y	f	complete body	deep frozen	deep frozen	NA	4.1% formalin
Callithrix jacchus	DPZ_CJ02	9y	f	complete body	deep frozen	deep frozen	NA	4.1% formalin
Cebuella pygmaea	AZ_CP_M10029	2y	f	right foot	ethanol 70%	ethanol 70%	NA	4.1% formalin
Cebuella pygmaea	AZ_CP_M11128	4y 11m	m	right foot	ethanol 70%	ethanol 70%	NA	4.1% formalin
Tupaia belangeri	TiHo_TB01	6y 5m	f	right foot	deep frozen	deep frozen	NA	4.1% formalin
Tupaia belangeri	TiHo_TB02	5y 7m	m	right foot	deep frozen	deep frozen	NA	4.1% formalin
Tamiops swinhoei	HUB_TS01	6y	m	complete body	deep frozen	ethanol 70%	2 months	4.1% formalin
Tamiops swinhoei	HUB_TS02	unknown	m	complete body	deep frozen	deep frozen	NA	4.1% formalin
Micromys minutus	ZL_MYM01	5m	m	complete body	deep frozen	deep frozen	NA	4.1% formalin
Micromys minutus	ZH_MYM02	1y 6m	m	complete body	deep frozen	deep frozen	NA	4.1% formalin
Petaurus sp.	ZMB-Mam_108791	unknown	unknown	right foot	ethanol 70%	ethanol 70%	NA	4.1% formalin
Petaurus breviceps	ZMB-Mam_108789	unknown	f	right foot	ethanol 70%	ethanol 70%	NA	4.1% formalin
Acrobates pygmaeus	ZMB-Mam_60330	unknown	unknown	right foot	ethanol 70%	ethanol 70%	NA	4.1% formalin
Monodelphis domestica	MfN_MD01	1y 4m	m	complete body	deep frozen	deep frozen	NA	4.1% formalin
Monodelphis domestica	MfN_MD02	2y 5m	f	complete body	deep frozen	deep frozen	NA	4.1% formalin
Marmosa sp.	MfN_MA01	unknown	unknown	right foot	ethanol 70%	ethanol 70%	NA	4.1% formalin
Marmosa sp.	MfN_MA02	unknown	unknown	right foot	ethanol 70%	ethanol 70%	NA	4.1% formalin
Gracilinanus sp.	MfN_GR01	unknown	unknown	right foot	ethanol 70%	ethanol 70%	NA	4.1% formalin

[Table S4 continued]

Species	Specimen	Days in fixative	Stain	Days in stain	Scanner	Scanning location	kV
Microcebus lehilahytsara	TiHo_ML01	1	1% iodine	3	SkyScan2211	MPI Science of Human History Jena	50
Microcebus lehilahytsara	TiHo_ML02	1	1% iodine	3	SkyScan2211	MPI Science of Human History Jena	50
Microcebus murinus	TiHo_MMU01	1	1% iodine	3	SkyScan2211	MPI Science of Human History Jena	50
Microcebus murinus	TiHo_MMU02	1	1% iodine	3	SkyScan2214	Prüflabor Bruker Karlsdorf-Neuthard	60
Callithrix jacchus	ZW_CJ01	1	1% iodine	4	YXLON FF20	Yxlon Hamburg	70
Callithrix jacchus	DPZ_CJ02	1	1% iodine	4	YXLON FF20	Yxlon Hamburg	80

Cebuella pygmaea	AZ_CP_M10029	52	1% iodine	7	SkyScan2211	MPI Science of Human History Jena	70
Cebuella pygmaea	AZ_CP_M11128	52	1% iodine	7	SkyScan2211	MPI Science of Human History Jena	60
Tupaia belangeri	TiHo_TB01	1	1% iodine	4	Zeiss Xradia Context	Jena	NA
Tupaia belangeri	TiHo_TB02	1	1% iodine	4	YXLON FF20	Yxlon Hamburg	80
Tamiops swinhoei	HUB_TS01	1	1% iodine	3	SkyScan2211	MPI Science of Human History Jena	40
Tamiops swinhoei	HUB_TS02	1	1% iodine	3	SkyScan2211	MPI Science of Human History Jena	50
Micromys minutus	ZL_MYM01	1	1% iodine	2	SkyScan2211	MPI Science of Human History Jena	50
Micromys minutus	ZH_MYM02	1	1% iodine	2	YXLON FF20	HU Berlin Verlg Zoologie	45
Petaurus sp.	ZMB-Mam_108791	2	1% iodine	5	SkyScan2211	MPI Science of Human History Jena	50
Petaurus breviceps	ZMB-Mam_108789	1	1% iodine	5	YXLON FF20	Yxlon Hamburg	70
Acrobates pygmaeus	ZMB-Mam_60330	1	1% iodine	5	YXLON FF20	Yxlon Hamburg	70
Monodelphis domestica	MfN_MD01	1	1% iodine	3	SkyScan2211	MPI Science of Human History Jena	50
Monodelphis domestica	MfN_MD02	1	1% iodine	3	SkyScan2211	MPI Science of Human History Jena	50
Marmosa sp.	MfN_MA01	1	1% iodine	5	YXLON FF20	Yxlon Hamburg	80
Marmosa sp.	MfN_MA02	1	1% iodine	5	YXLON FF20	Yxlon Hamburg	80
Gracilinanus sp.	MfN_GR01	1	1% iodine	5	YXLON FF20	Yxlon Hamburg	80

[Table S4 continued]

**Species**	**Specimen**	**microA**	**Voxel size (µm)**	**File format**	**Depth (bits)**	**Overall scan time**	**Image stack (slice number)**
Microcebus lehilahytsara	TiHo_ML01	270	3x3x3	TIFF	16	14h 14min	11050
Microcebus lehilahytsara	TiHo_ML02	300	3.5x3.5x3.5	TIFF	16	13h 01min	8837
Microcebus murinus	TiHo_MMU01	300	3.3x3.3x3.3	TIFF	16	13h 51min	8375
Microcebus murinus	TiHo_MMU02	139	6.0007x6.0007x6.0007	PNG	16	6h 23min	5333
Callithrix jacchus	ZW_CJ01	100	11x11x11	TIFF	NA	5h	5401
Callithrix jacchus	DPZ_CJ02	100	11x11x11	TIFF	NA	5h	4901
Cebuella pygmaea	AZ_CP_M10029	300	3.2x3.2x3.2	JPG	16	11h 55min	10911
Cebuella pygmaea	AZ_CP_M11128	280	3.5x3.5x3.5	PNG	16	11h 40min	10665
Tupaia belangeri	TiHo_TB01	NA	9.0442x9.0442x9.0442	TIFF	NA	NA	4715
Tupaia belangeri	TiHo_TB02	100	6x6x6	TIFF	NA	5h 5min	6300
Tamiops swinhoei	HUB_TS01	180	5.13x5.13x5.13	JPG	16	11h 23min	6226
Tamiops swinhoei	HUB_TS02	200	7x7x7	JPG	16	09h 06min	4671
Micromys minutus	ZL_MYM01	270	2.6x2.6x2.6	TIFF	16	08h 14min	6745
Micromys minutus	ZH_MYM02	145	4.47x4.47x4.47	TIFF	16	02h 07min	1867
Petaurus sp.	ZMB-Mam_108791	300	3.6x3.6x3.6	TIFF	16	10h 21min	8763
Petaurus breviceps	ZMB-Mam_108789	100	7x7x7	TIFF	NA	5h	4750
Acrobates pygmaeus	ZMB-Mam_60330	100	4x4x4	TIFF	NA	5h	4445
Monodelphis domestica	MfN_MD01	300	3.5x3.5x3.5	TIFF	16	NA	6769
Monodelphis domestica	MfN_MD02	300	3.5x3.5x3.5	TIFF	16	7h	4198
Marmosa sp.	MfN_MA01	100	7x7x7	TIFF	NA	NA	1793
Marmosa sp.	MfN_MA02	100	7x7x7	TIFF	NA	NA	2273
Gracilinanus sp.	MfN_GR01	100	5x5x5	TIFF	NA	NA	1839

Figure S1. Joint morphology in *Microcebus* (strepsirrhine primates)
Morphology of the entocuneiform-metatarsal joint exemplified in a foot model (ventral view) in one specimen of **A** *Microcebus lehilahytsara* and **B** *Microcebus murinus*. "Lateral" and "medial" are views of the entocuneiform and metatarsal; "proximal" is a view of the metatarsal's proximal articular surface, and "distal" is a view of the entocuneiform's distal articular surface. This figure is related to Figure 5.

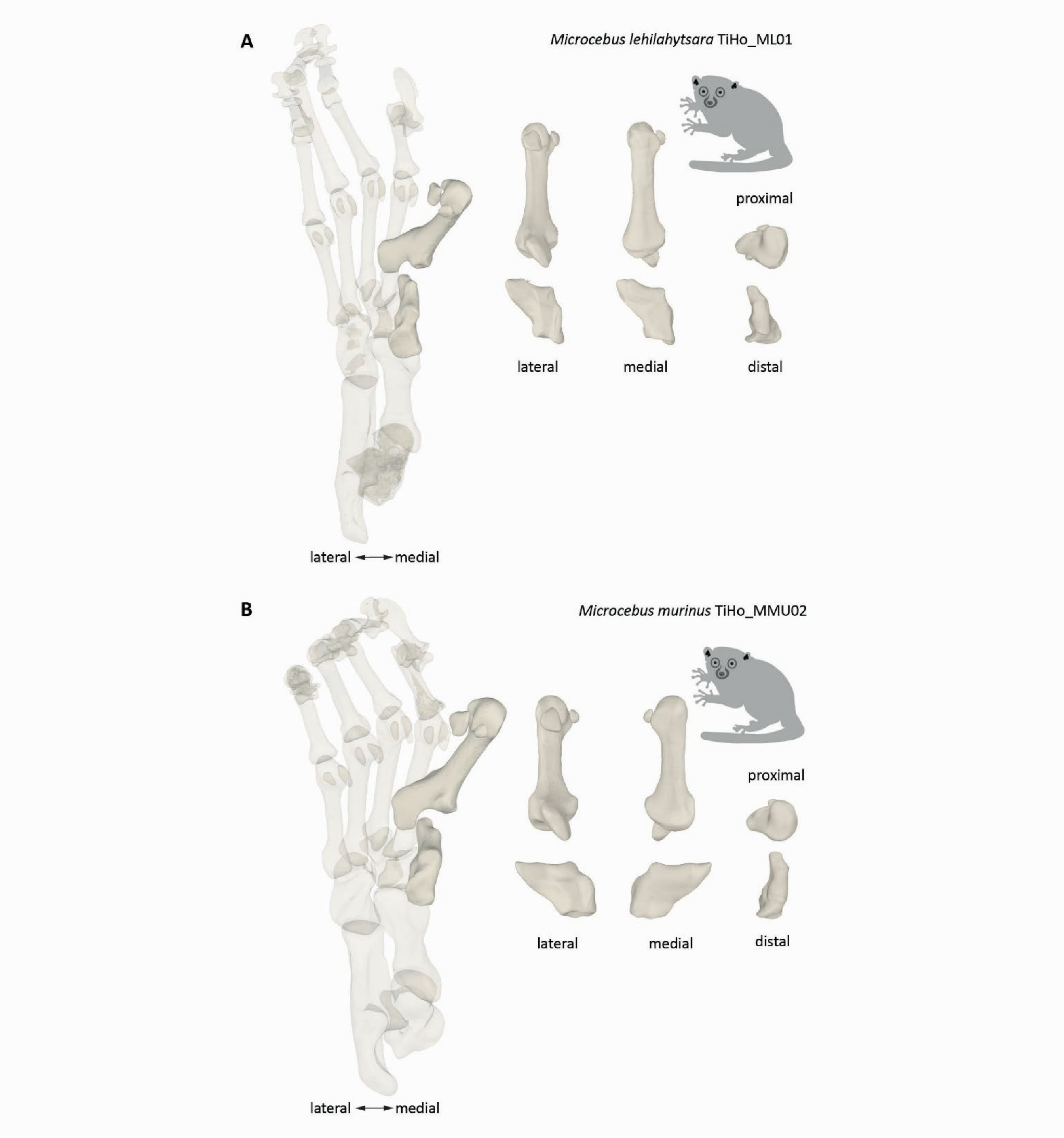

Figure S2. Joint morphology in callitrichid primates
Morphology of the entocuneiform-metatarsal joint exemplified in a foot model (ventral view) in one specimen of **A** *Cebuella pygmaea* and **B** *Callithrix jacchus*. “Lateral” and “medial” are views of the entocuneiform and metatarsal; “proximal” is a view of the metatarsal's proximal articular surface, and “distal” is a view of the entocuneiform's distal articular surface. This figure is related to Figure 5.

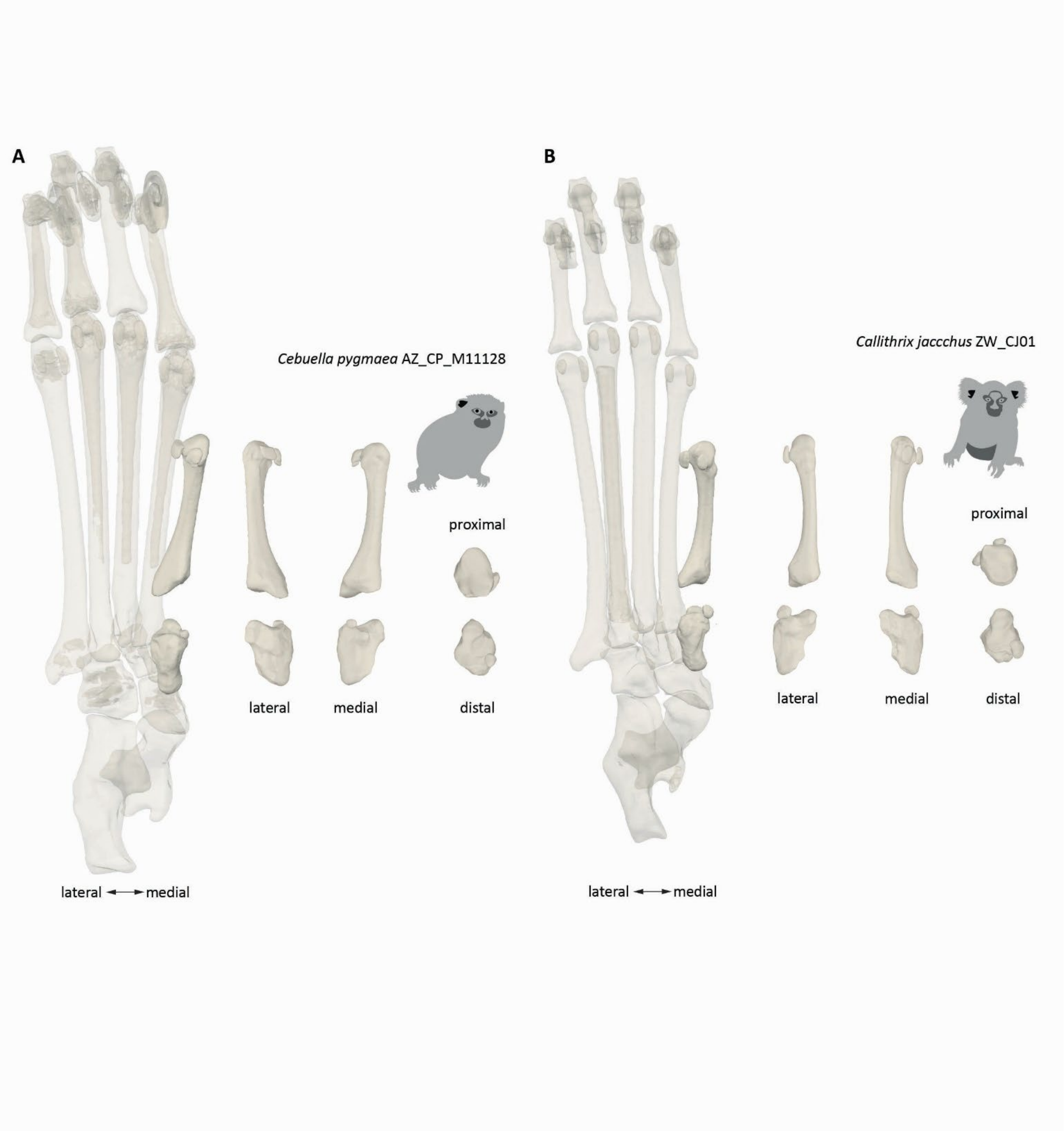

Figure S3. Joint morphology in rodents
Morphology of the entocuneiform-metatarsal joint exemplified in a foot model (ventral view) in one specimen of **A** *Tamiops swinhoei* and **B** *Micromys minutus*. “Lateral” and “medial” are views of the entocuneiform and metatarsal; “proximal” is a view of the metatarsal's proximal articular surface, and “distal” is a view of the entocuneiform's distal articular surface. This figure is related to Figure 5.

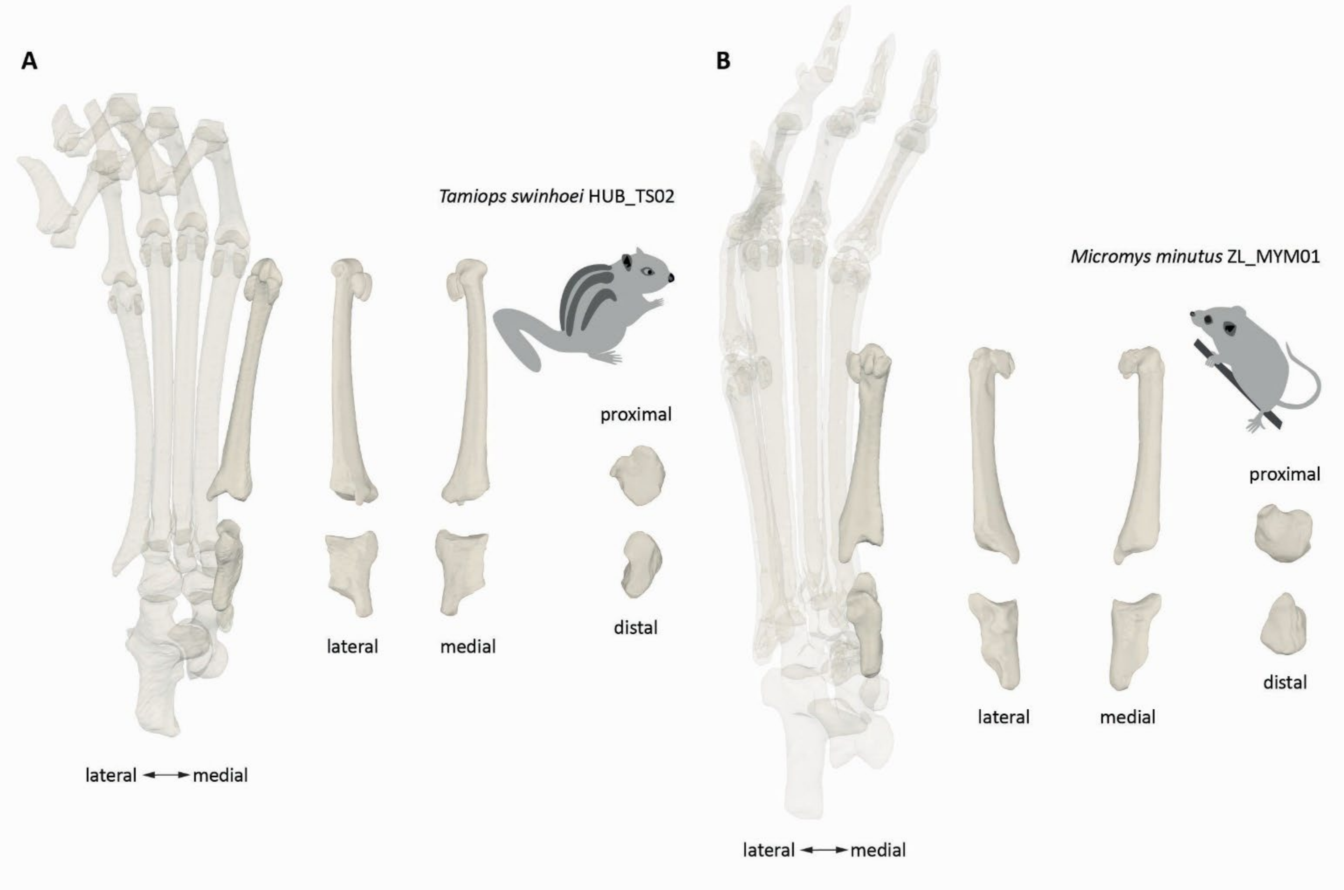

Figure S4. Joint morphology in *Tupaia belangeri* (scandentian) and *Monodelphis domestica* (didelphid marsupial)
Morphology of the entocuneiform-metatarsal joint exemplified in a foot model (ventral view) in one specimen of **A** *Tupaia belangeri* and **B** *Monodelphis domestica*. "Lateral" and "medial" are views of the entocuneiform and metatarsal; "proximal" is a view of the metatarsal's proximal articular surface, and "distal" is a view of the entocuneiform's distal articular surface. This figure is related to Figure 5.

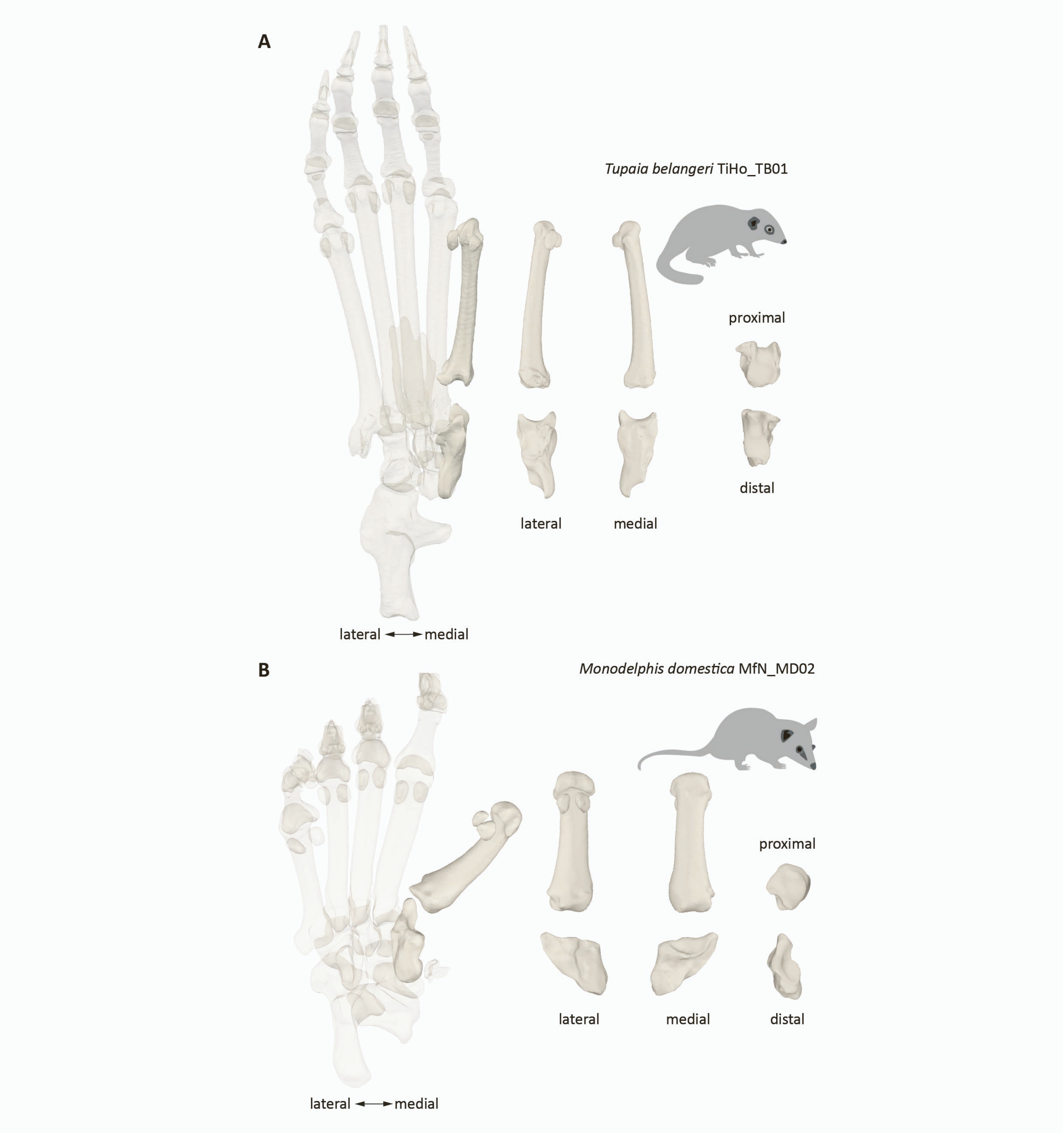

Figure S5. Joint morphology in didelphid marsupials
Morphology of the entocuneiform-metatarsal joint exemplified in a foot model (ventral view) in one specimen of **A** *Marmosa* sp. and **B** *Gracilinanus* sp. “Lateral” and “medial” are views of the entocuneiform and metatarsal; “proximal” is a view of the metatarsal's proximal articular surface, and “distal” is a view of the entocuneiform's distal articular surface. This figure is related to Figure 5.

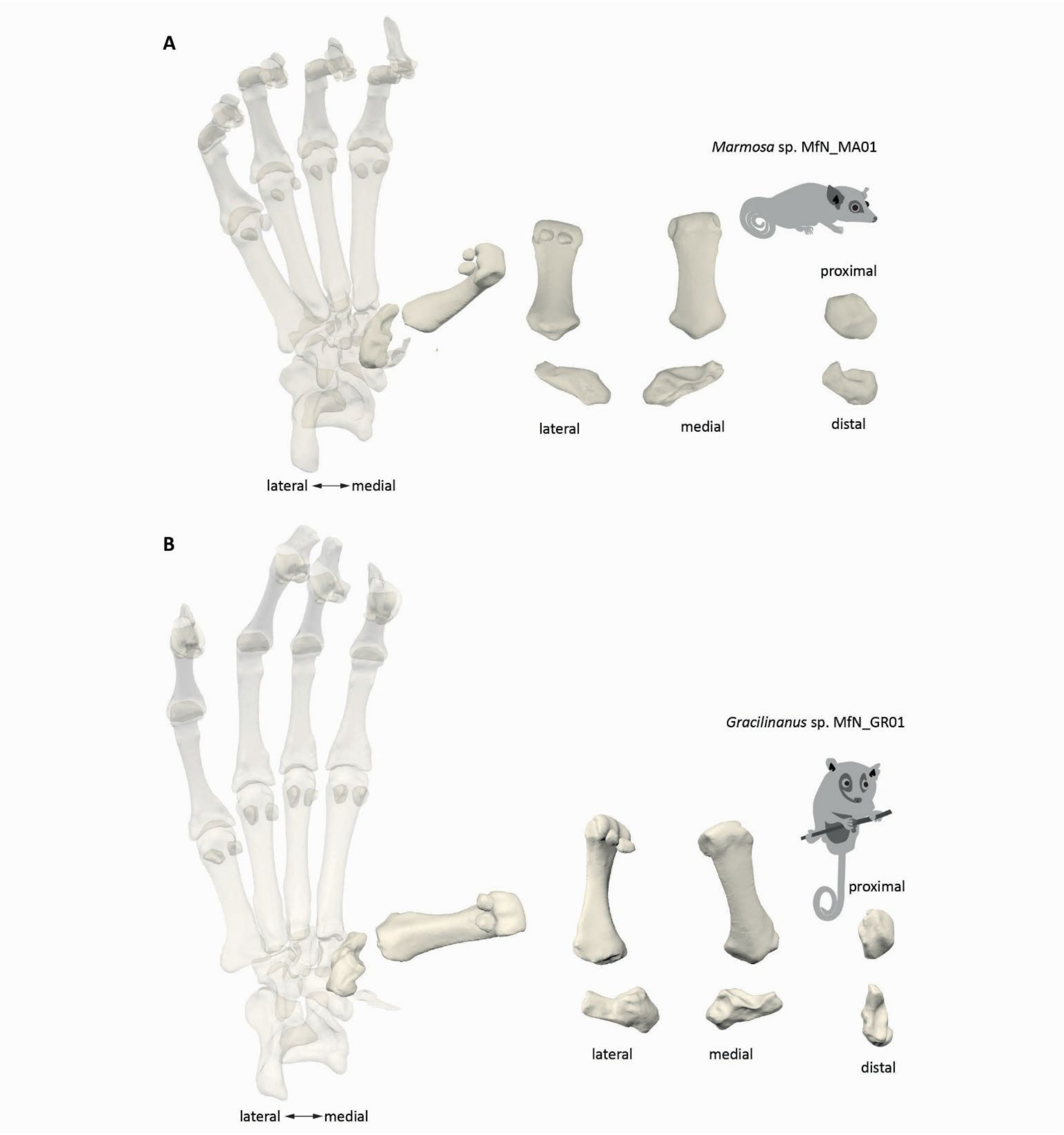

Figure S6. Joint morphology in diprotodontian marsupials
Morphology of the entocuneiform-metatarsal joint exemplified in a foot model (ventral view) in one specimen of **A** *Petaurus* sp. and **B** *Acrobates pygmaeus*. “Lateral” and “medial” are views of the entocuneiform and metatarsal; “proximal” is a view of the metatarsal's proximal articular surface, and “distal” is a view of the entocuneiform's distal articular surface. This figure is related to Figure 5.

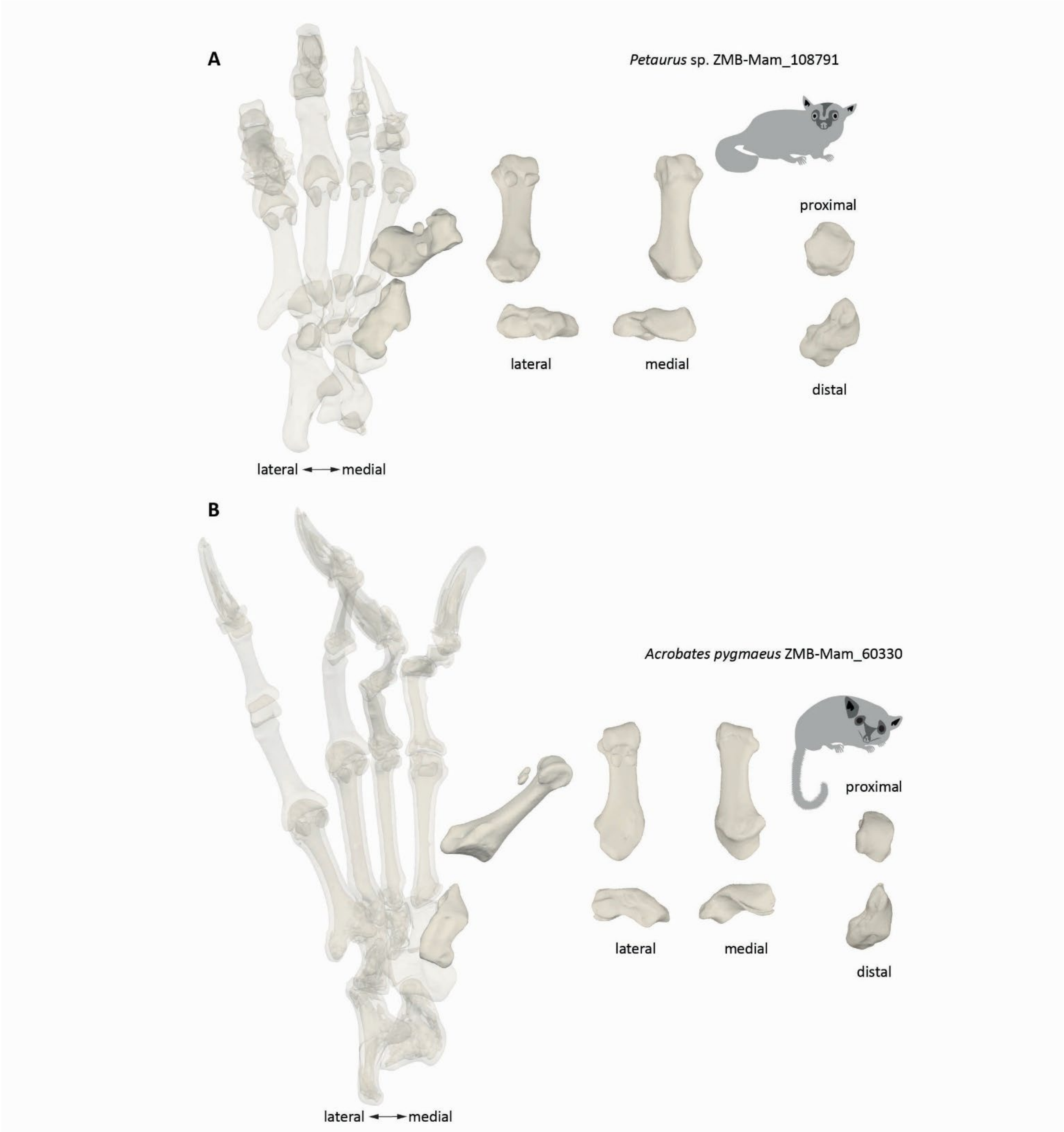

Figure S7. Linear relationships of architectural variables and body mass
The investigated muscular architectural variables of volume (Vol_cf) and anatomical cross-sectional area (ACSA_cf) are plotted against body mass using a logarithmic scale for the **A** adductor hallucis, the transverse head of the adductor hallucis (T), the oblique head of the adductor hallucis (O), and **B** the flexor hallucis brevis. Slopes and 95% confidence interval (CI) of linear regressions in correlations of volume and ACSA with body mass are reported. This Figure is related to STAR Methods.

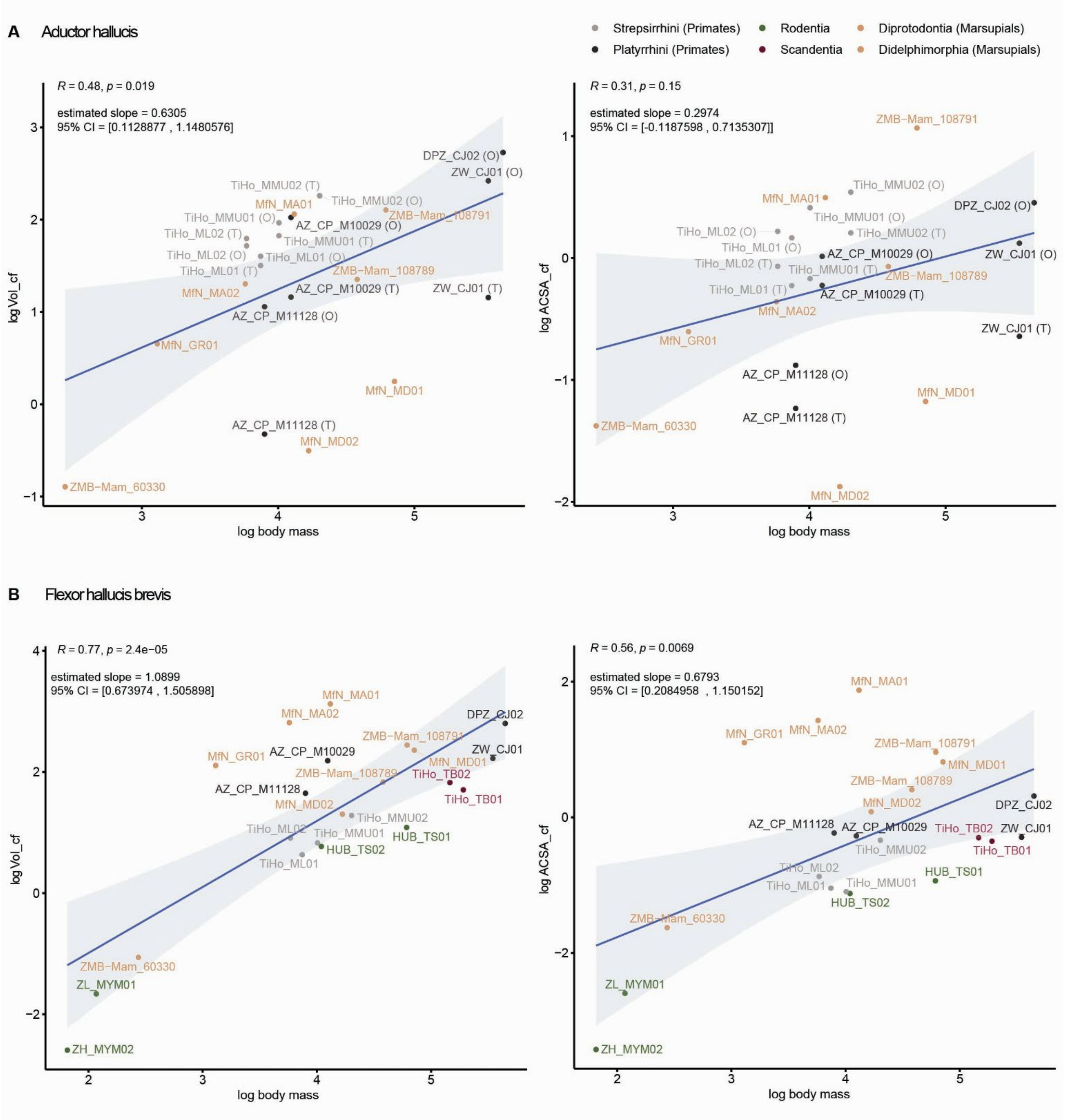

Supplement: Document S1. Figures S1–S7 and Tables S3, and S4 [file mmc1.pdf]
